# Trends in food and nutrient intake over 20 years: findings from the 1998-2018 Korea National Health and Nutrition Examination Survey

**DOI:** 10.4178/epih.e2021027

**Published:** 2021-04-19

**Authors:** Sanghui Kweon, Jin Young Park, Myungsook Park, Yangha Kim, So Yeong Yeon, Leena Yoon, Sungha Yun, Suyeon Park, Ji Eun Yang, Youngtaek Kim, Ok Park, Kyungwon Oh

**Affiliations:** 1 Division of Health and Nutrition Survey and Analysis, Bureau of Chronic Disease Prevention and Control, Korea Disease Control and Prevention Agency, Cheongju, Korea; 2 Public Health Medical Service Office, Chungnam National University Hospital, Daejeon, Korea

**Keywords:** Korea National Health and Nutrition Examination Survey, Food intake, Nutrient intake, Nutritional status

## Abstract

**OBJECTIVES:**

We aimed to examine the current status and trends of food and nutrient intake in the Korean population over the past 20 years using the data from the Korea National Health and Nutrition Examination Survey (KNHANES).

**METHODS:**

We conducted a survey of 116,284 subjects over the age of one year in Korea, who participated in the KNHANES between 1998 and 2018. We collected data on the subjects’ intake for the day before using the 24-hour recall method. The annual percent change (APC) in the food groups and nutrient intake were calculated using SAS and Joinpoint software.

**RESULTS:**

The intake of grains (APC=-0.4, p<0.05) and vegetables (APC=-0.8, p<0.05) was observed to decrease. In contrast, the intake of beverages, meat, dairy, and eggs increased. In particular, beverage intake increased by more than four times (APC=9.2, p<0.05). There was no significant change in energy intake. However, the proportion of energy intake from carbohydrates decreased by approximately 5%p (APC=-0.3, p<0.05), whereas that from fat increased by approximately 5%p (APC=1.1, p<0.05). Additionally, there were decreases in the proportion of energy intake from breakfast and homemade meals and increases in the energy intake from snacks, dining out, and convenience food. The intake of vitamin C (APC=-3.2, p<0.05) and sodium (APC=-2.3, p<0.05) significantly decreased.

**CONCLUSIONS:**

Over the past 20 years, there has been decreases in the intake of grains, vegetables, carbohydrates, sodium, and vitamin C and increases in the intake of beverages, dairy, meat, eggs, and fat. Since nutritional status is an important factor in the prevention and management of chronic diseases, it should be continuously monitored.

## INTRODUCTION

According to the Global Burden of Disease Study (GBD), one in five deaths can be prevented through an improvement in diet [1]. However, while types of food that are recommended for the prevention of chronic diseases (i.e., whole grains, milk, and nuts) are consumed lesser than the recommended level, the types of food that are not recommended (i.e., sodium, sweetened beverages, and processed meat) tend to be consumed in a larger quantity. For these reasons, GBD suggested that it is necessary to improve the nutritional intake to reduce the number of deaths and the burden of disease caused by chronic diseases. In Korea, nutritional factors, including excess sodium intake and insufficient intake of dietary fiber and whole grains, account for the third leading risk factor contributing to death after smoking and hyperglycemia [2].

Nutrition is an important factor in the prevention and management of chronic diseases, such as cardiovascular disease, cancer, and diabetes. In most countries, including Korea, a national-level survey has been conducted to determine the changes in the nutritional intake with the aim of preventing and managing chronic diseases [3,4]. In Korea, the Korea National Health and Nutrition Examination Survey (KNHANES) is used to evaluate the nutrition policies by monitoring the nutritional status, identifying nutritionally vulnerable groups, and comparing the targets for the objectives of the National Health Plan (HP) [5].

In Korea, deaths due to chronic diseases, such as cancer, cardiovascular disease, and diabetes account for approximately 80% of all the deaths [6]. Over the past 20 years, the prevalence of obesity and hypercholesterolemia has increased, while the prevalence of hypertension and diabetes has stagnated [7]. Therefore, it is necessary to prepare a prevention and management plan through an in-depth understanding of the changes in the nutritional intake, which are the major risk factors for chronic diseases. Although the Korea Disease Control and Prevention Agency (KDCA, formerly the Korea Centers for Disease Control and Prevention) publishes a report (“*Health Statistics*”) every year, it is not enough to describe the results for the trends in the nutritional intake, because it also includes results from health examination and health interview. Some previous studies [8,9] have reported changes in the diet of Koreans, such as an increased intake of animal food groups and fat. However, since these studies were based on the data collected prior to 1998, it is necessary to understand the latest changes in the diet. While there are other previous studies [10,11] that analyzed more recent data, it remains difficult to understand the changes in the overall nutritional status, as their results are mainly concerned with particular aspects of nutrients, such as energy and sodium.

This study aimed to examine the changes in the intake of major foods and nutrients over the past 20 years using the data from the KNHANES (1998-2018) to provide evidence for the prevention and management of chronic diseases in Korea.

## MATERIALS AND METHODS

### Study subjects

Based on the National Health Promotion Act enacted in 1995, the KNHANES has been conducted since 1998 for the production of national health statistics. KNHANES was conducted in November-December in 1998 and 2001, April-June in 2005 (April-May nutrition survey), and July-December in 2007. From 2008, it has been conducted as an annual survey (January-December) to produce statistics without seasonal variation. Since 1998, a two-stage stratified cluster sampling method was selected for approximately 200 primary sampling units (PSU) and 20 households to 23 households per PSU. All eligible members aged one year and above within the sample households become the target sample.

Subjects were all over one-year-old and members of the household that was sampled using the method described above. For our analysis, we used the data from 116,284 people over one year of age who completed the 24-hour dietary recall between 1998 (first survey) and 2016-2018 (seventh survey).

### Nutrition survey

The KNHANES consists of a health examination, a health interview, and a nutrition survey. The nutrition survey is divided into a 24-hour dietary recall, a dietary behavior survey, and a food security survey.

For the 24-hour dietary recall, a team of dieticians visited each subject’s household and conducted individual interviews with all the household members over the age of one year to collect data about the name and amount consumed of the dish or food as well as the location and type of meal eaten during the day before in chronological order [12]. To determine the exact amount of the intake, we investigated each individual’s intake using various measuring aids. If a subject had eaten dishes cooked in the household, the ingredients and their amounts used for cooking the meals were surveyed and reflected on their personal food and nutrient intake. When the subjects dined out, we used the recipe database (DB), which is composed of the ingredient list of a dish and the amount of each food ingredient, to calculate the intake of food, and the nutrients from the dishes consumed. For each individual’s daily intake, the energy and nutrient intakes were calculated using the nutrient DB for each food established based on the National Standard Food Composition Table [7].

The main food groups examined in our study, which showed differences in the amount of intake across years, were grains, vegetables, beverages (non-alcoholic beverages), fruits, meat, dairy (milk and dairy products), and eggs. For nutrients, we examined the total energy and the proportion of energy from carbohydrates, proteins, and fats and the components for the fourth HP objective, including vitamin A, riboflavin, vitamin C, calcium, sodium, and iron. To examine the changes in the energy composition, we presented the proportion of energy intake for the source nutrients of energy: fat, carbohydrate, and protein. As the unit of reference intake for vitamin A changed from μg RE to μg RAE in the Dietray Reference Intakes for Koreans 2015 (2015 KDRIs), KNHANES reported the vitamin A intake in μg RAE from the seventh survey (2016-2018). In this study, we used the unit μg RE for vitamin A intake so that we could compare the data over the past 20 years. The proportion of energy intake from each meal and meal type was also examined to identify the dietary changes. The meals were divided into breakfast, lunch, dinner, and snacks. The meal types were divided into homemade meals, dining out (dish from a restaurant or an institutional food service), single food (i.e., fruit, snack, and milk), and convenience food (ready-to-eat food or ready-to-cook food).

### Statistical analysis

All analyses were performed using SAS version 9.4 (SAS Institute Inc., Cary, NC, USA) and Joinpoint Regression Program version 4.1.1.1 (US National Cancer Institute [NCI], Bethesda, MD, USA). To represent the Korean population, the sampling weights assigned to subjects were applied to all analyses. The sampling weights were generated by considering a complex sample design, non-response rate of the target population, and post-stratification. To adjust for differences in the results from changes in the age structure of each year, age-standardized results were calculated using the age- and sex-specific structures of the estimated population based on the 2005 population projections for Korea. The estimates and their standard errors obtained from SAS were input into NCI’s Joinpoint program, which was estimated by setting the joinpoint as 0 or 1, and the annual percent change (APC) was calculated. APC verified that the annual rate of change was “0” under the significance level of 0.05; additionally, the Monte Carlo method in Joinpoint Regression Program was used to test the statistical significance of the optimal.

### Ethics statement

This study was approved by the Institutional Review Board of the KDCA (2007–2014, 2018). For certain year (2015-2017), ethical approval was waived by the Act (Article 2, Paragraph 1) and Enforcement Regulation (Article 2, Paragraph 2, item 1) of Bioethics and Safety Act.

## RESULTS

A total of 116,284 subjects (52,213 males, 64,071 females) over the age of one year, who had completed the 24-hour dietary recall of KNHANES (1998-2018) were included. Their average age increased by about ten years, from 34.5 to 43.5 years over the past 20 years. The proportion of subjects who were college graduates or higher increased by approximately 10%p from 2005 to 2018 (Table 1).

Since KNHANES was introduced in 1998, the intake of grains has decreased (APC=-0.4, p<0.05). In particular, the intake of grains by female subjects significantly decreased (Table 2). The intake of vegetables has decreased since 2005 (APC=-1.5, p<0.05), whereas the intake of fruits showed a tendency to decrease, but not to a statistically significant level. The food group with the largest change in intake over the past 20 years was beverages, which increased significantly since 2005 to become 4.6 times more than the intake in 1998 (45.3 g in 1998 and 208.4 g in 2018; APC=9.2, p<0.05). During the same period, meat intake was also observed to increase. In particular, the intake of meat by male subjects doubled over the past 20 years (82.7 g in 1998 and 160.0 g in 2018; APC=0.7, p<0.05). The intake of dairy tended to increase continuously until 2011 (APC=3.6, p<0.05); however, there was no significant change afterward. The intake of eggs increased, resulting in an intake of 31.0 g in 2018, 1.5 times that of 21.7 g in 1998 (APC=2.0, p<0.05).

The total energy intake tended to increase over the past 20 years, but only by a significant amount in males (APC=0.7, p<0.05) (Table 3). The proportion of the energy intake from fat increased significantly from 17.9% in 1998 to 22.6% in 2018 (APC =1.1, p<0.05). Furthermore, this increase has accelerated in both male and female subjects since 2009. During the same period, the proportion of energy intake from carbohydrates decreased by 4.9%p (67.1% in 1998 and 62.2% in 2018), whereas that from protein did not significantly change. The proportion of energy intake from breakfast significantly decreased (23.1% in 1998 and 16.2% in 2018; APC=-1.8, p<0.05), whereas that from snacks increased (APC=1.5, p<0.05). While the proportion of energy intake from homemade meals decreased, that from dining out almost doubled (18.9% in 1998 and 36.6% in 2018), and that from food or convenience food increased by approximately 1.5 times (15.5% in 1998 and 25.1% in 2018).

The intake of vitamin C and sodium significantly decreased, whereas riboflavin intake significantly increased (Table 4). The decreasing trend of vitamin C intake accelerated from 2014 (APC=-11.7, p<0.05). The sodium intake decreased significantly over 20 years (4,585.6 mg in 1998 and 3,255.0 mg in 2018), particularly since 2010 (APC=-4.9, p<0.05). The vitamin A intake tended to increase until 2011 (APC=2.4, p<0.05), but decreased thereafter (APC=-5.3, p<0.05). There was no change in the calcium intake, whereas the iron intake tended to decrease in 2014 (APC=-8.1, p<0.05).

## DISCUSSION

Since the introduction of the KNHANES in 1998, the intake of grains, vegetables, and fruits has decreased, whereas the intake of beverages, meat, dairy, and eggs has increased in the past 20 years. Additionally, these changes were related to the changes in the nutrient intake, resulting in a decrease in the intake of vitamin C and increase in the intake of riboflavin. The total energy intake of the male subjects tended to increase slightly. The proportion of energy intake from fat increased; similarly, the energy intake from dining out or convenience food increased.

In our study, the intake of plant-based foods and the proportion of energy from carbohydrates decreased, whereas the intake of animal-based foods and the proportion of energy from fat increased. The increase in the intake of animal food and fat was consistent with the results reported in previous domestic studies, such as a study that analyzed the changes in the food intake from 1969 to 1995, which suggests that there was a tendency to change from before 1998 [8,9]. The composition of the households in Korea has also changed; for example, the number of single-person households has increased [13]. Furthermore, more females are now employed [14], leading to changes in the food environment. Upon reviewing the energy intake trends for each meal and type of meal, we found changes in the diet, such as a decreased energy intake from breakfast and homemade meals, and an increase in the proportion of energy intake from snacks, dining out, and convenience food. These changes in the diet are believed to have contributed to changes in the sources of energy intake.

The food group with the largest changes in the intake over the past 20 years was beverages. While the year in which jointpoints occurred differed between male and female subjects, both of their intakes significantly increased. As sugar-sweetened beverages (SSB) contribute to a large proportion of the energy and total sugar intake, it was recommended to reduce the consumption of SSB as much as possible [15,16]. The energy intake from beverages in subjects over one-year-old in Korea increased by 41.8 kcal (30.7 kcal in 1998 and 72.5 kcal in 2018), which may have contributed to the increase in the total energy intake. In addition, beverages, such as sugar-added coffee, soft drinks, and fruit-based beverages were considered as major sources of total sugar intake [7]. Further research that analyzes the trend of beverage intake, which divides beverages into subclasses (sweetened or unsweetened) is needed.

In contrast, the intake of vegetables has decreased significantly over the past 20 years. While the intake of fruits showed a decreasing tendency during the same period, this change was not statistically significant. Considering that fruits and vegetables may have different intakes depending on the season compared to other food groups, we analyzed their APC between 2008, the year in which the annual survey was introduced, and 2018. We found that the intake of fruits has decreased in both male and female subjects by approximately 11% every year since 2015. Moreover, the intake of vegetables has greatly decreased since 2014 compared to 2008-2013 (data not shown). The proportion of adults over the age of 19 years, who consumed more than 500 g of fruits and vegetables decreased from 42.9% in 1998 to 29.4% in 2018. It particularly decreased significantly in those aged 19-29 years (decrease by 25.1%p) and 30-49 years (decrease by 19.7%p). The cause of this decrease in young and middle-aged adults should be further examined [7].

The energy intake was 1,988 kcal in 2018 (aged ≥ 1 years), which has slightly increased (53 kcal) over the past 20 years. In particular, the male subjects were found to have a significant increase in energy intake (149 kcal). There was no significant change in the energy intake in female subjects over the past 20 years; however, the APC over the ten-year period from 2008 to 2018 showed a tendency to increase (APC=0.7, p<0.05; data not shown). The energy intake was 2,093 kcal in the United States (2017-2018, aged ≥ 2 years) [3] and 1,900 kcal in Japan (2018, aged ≥ 1 years), which did not differ significantly from the energy intake in Korea [4]. However, unlike the energy intake trends in Korea, the energy intake in Japan has tended to decrease over the past 20 years (1995-2016) [17].

Although there was no significant change in the energy intake, the proportion of energy intake from carbohydrates decreased in both male and female subjects. The proportion of energy intake from fat increased by 4.7%p over the past 20 years, reaching 22.6% (fat intake=49.5 g) in 2018 [7]. This increase was statistically significant in both male and female subjects. Similar trends were also reported in studies conducted in the United States and Japan [17,18]. The proportion of energy intake from fat in Korea was lower than that in the United States (36.0%; fat intake=85.0 g) and Japan (28.3%; fat intake=60.4 g). Furthermore, it fell within the acceptable macronutrient distribution range of the 2015 KDRIs. However, it has increased by approximately 5%p over 20 years, especially 4%p over the last ten years. The proportion of people who consume more than 30% of the total energy from fat has also increased. In particular, younger age groups, such as adults in their 20s (14% in 1998 and 29% in 2018) and 30s (9% in 1998 and 24% in 2018) showed significant increases (data not shown). Given these trends and the increasing prevalence of hypercholesterolemia and obesity, it is necessary to continuously monitor the intake of fat and the proportion of energy intake from fat.

The nutrient intake was influenced by changes in food intake. The decreased consumption of fruits and vegetables may have contributed to a decrease in the vitamin C intake, whereas increases in the consumption of meat, dairy, and eggs may have contributed to an increase in the riboflavin intake. The intake of calcium has remained fairly unchanged over the past 20 years, as the intake of dairy has increased and the intake of vegetables has decreased. The nutrient with the greatest change in intake was sodium, which showed statistically significant decreases in male and female subjects. The decrease in the sodium intake may have been caused by factors, such as a decrease in the intake of major food sources and the enforcement of policies to reduce the sodium intake. The intake of cabbage kimchi, a major source of sodium for Koreans, decreased from 83.8 g in 1998 to 62.9 g in 2018 [7,19]. In addition, as the need for sodium reduction emerged in Korea, a national task force established by the government in 2007 and the National Plan to Reduce Sodium, including a campaign to improve public awareness and voluntary reformulation of processed foods, such as fried noodles, paste, and confectionery to lower the sodium content, was implemented in 2012 [20]. Consequently, the sodium intake was significantly reduced to 3,255 mg in 2018 compared to 4,586 mg in 1998. However, considering that it is still higher than the recommended maximum level of 2,000 mg and that about 75% of people over the age of nine years consume 2,000 mg or more, further initiatives are still required to reduce the intake of sodium [7].

While the 24-hour dietary recall of KNHANES was conducted using the same method for 20 years, there were differences in the survey period and the nutritional DB used to calculate the results. For example, the average intake of fruits showed a difference of more than 100 g depending on the survey period: 197.3 g in 1998 and 208.3 g in 2001 (survey period from November to December) and 87.6 g in 2005 (survey period from April to May) [7]. This suggests that seasonal changes may have influenced the intake of fruits and vegetables. Accordingly, we performed a further analysis of the food and nutrient intake trends from 2008, the year in which the annual survey was established to 2018. While we observed no significant change in fruits intake over the past 20 years, an analysis using the data between 2008 and 2018 showed a decreasing trend since 2015 (APC=-11.1, p<0.05). Unlike the 20-year analysis, the ten-year analysis showed significant changes in the energy intake of female subjects (APC=0.7, p<0.05), as well as the proportion of energy intake from protein (APC=0.4, p<0.05) and vitamin A intake (APC=-3.4, p<0.05). Other foods and nutrients showed slight differences in the APC; however, their increasing or decreasing tendencies were similar to those analyzed over the 20-year period (data not shown). Second, since the main purpose of the KNHANES is to estimate the nutritional status of the current year, it uses the latest recipe DB and nutrient DB for each food to calculate the results. This is beneficial because the results reflect the nutritional information when the nutritional status is evaluated. However, the effect of the new DB needs to be considered when evaluating the food and nutrient intake trends. For example, since iron intake calculated according to the National Standard Food Composition Table version 9.1 [21] was lower than that of the Revised version 8 [22] (data not shown), the difference between DBs needs to be considered when comparing the results between the sixth (2013-2015) and seventh (2016-2018) KNHANES. When changing the DB for data processing, we calculated the results by applying each of the existing DB and the new DB to the same 24-hour dietary recall data. Subsequently, these results were reviewed by the relevant government agencies and experts. For reference, the information on DB used to calculate the survey results is described in detail and published in the “*Health Statistics*” and the “*Guidebook for Data Users*” of KNHANES.

In conclusion, there have been few positive changes in food and nutrient intake except for a decrease in the sodium intake, such as the intake of fruits and vegetables decreased and the intake of beverages and fat increased over the past 20 years. Since the nutritional intake is an important factor in preventing and managing chronic diseases, it is necessary to develop and actively enforce nutrition policies to promote the nutritional status. In addition, since the KNHANES is an ongoing surveillance system that supports the development of health policies, it is necessary to improve the survey method and conduct in-depth analysis to explore the nutritional problems and nutritional factors related to chronic diseases.

## Figures and Tables

**Table 1.** Characteristics of subjects in the Korea National Health and Nutrition Examination Survey

Characteristics			1998	2001	2005	2007	2008	2009	2010	2011	2012	2013	2014	2015	2016	2017	2018
Total (n)			10,400	9,968	8,930	4,091	8,631	9,391	8,019	7,704	7,208	7,242	6,801	6,628	7,040	7,167	7,064
	Age (yr)		34.5±0.2	34.0±0.2	36.1±0.2	38.2±0.4	38.4±0.2	39.1±0.2	39.4±0.3	41.3±0.3	42.5±0.3	40.0±0.3	42.6±0.3	43.6±0.3	41.5±0.3	43.4±0.3	43.5±0.3
		1-18	27.9 (0.4)	28.9 (0.5)	26.9 (0.5)	28.2 (0.7)	27.3 (0.5)	25.7 (0.5)	25.9 (0.5)	23.6 (0.5)	22.8 (0.5)	24.9 (0.5)	21.7 (0.5)	19.9 (0.5)	22.9 (0.5)	20.2 (0.5)	19.3 (0.5)
		19-64	62.8 (0.5)	62.0 (0.5)	61.8 (0.5)	54.5 (0.8)	55.8 (0.5)	57.0 (0.5)	56.8 (0.6)	57.1 (0.6)	55.2 (0.6)	57.2 (0.6)	56.0 (0.6)	58.1 (0.6)	56.8 (0.6)	58.3 (0.6)	59.6 (0.6)
		≥65	9.4 (0.3)	9.2 (0.3)	11.2 (0.3)	17.4 (0.6)	16.9 (0.4)	17.3 (0.4)	17.3 (0.4)	19.3 (0.4)	22.0 (0.5)	17.9 (0.5)	22.2 (0.5)	22.0 (0.5)	20.4 (0.5)	21.6 (0.5)	21.1 (0.5)
	Residence																
		Urban	63.4 (0.5)	79.0 (0.4)	80.5 (0.4)	72.0 (0.7)	75.7 (0.5)	76.0 (0.4)	79.1 (0.5)	81.0 (0.4)	80.6 (0.5)	80.1 (0.5)	80.3 (0.5)	81.1 (0.5)	80.7 (0.5)	81.9 (0.5)	82.4 (0.5)
		Rural area	36.6 (0.5)	21.0 (0.4)	19.5 (0.4)	28.0 (0.7)	24.3 (0.5)	24.0 (0.4)	20.9 (0.5)	19.0 (0.4)	19.4 (0.5)	19.9 (0.5)	19.7 (0.5)	18.9 (0.5)	19.3 (0.5)	18.1 (0.5)	17.6 (0.5)
	Education																
		≤Elementary school	-	-	39.8 (0.5)	48.2 (0.8)	46.9 (0.6)	43.6 (0.5)	43.0 (0.6)	40.9 (0.6)	40.8 (0.6)	39.8 (0.6)	39.4 (0.6)	37.0 (0.6)	37.2 (0.6)	35.7 (0.6)	33.0 (0.6)
		Middle school	-	-	11.8 (0.3)	10.6 (0.5)	11.2 (0.3)	12.0 (0.3)	10.9 (0.4)	11.7 (0.4)	11.1 (0.4)	11.2 (0.4)	11.6 (0.4)	11.6 (0.4)	10.5 (0.4)	10.8 (0.4)	10.2 (0.4)
		High school	-	-	29.3 (0.5)	23.9 (0.7)	24.6 (0.5)	26.0 (0.5)	24.1 (0.5)	25.4 (0.5)	25.7 (0.5)	26.2 (0.5)	25.0 (0.6)	26.5 (0.6)	24.5 (0.5)	24.2 (0.5)	26.7 (0.5)
		≥College	-	-	19.0 (0.4)	17.2 (0.6)	17.3 (0.4)	18.4 (0.4)	22.0 (0.5)	22.0 (0.5)	22.4 (0.5)	22.8 (0.5)	24.1 (0.6)	25.0 (0.6)	27.8 (0.6)	29.3 (0.6)	30.1 (0.6)
Male (n)			4,984	4,760	4,167	1,821	3,692	4,182	3,550	3,376	3,127	3,196	2,976	2,942	3,063	3,233	3,144
	Age (yr)		33.1±0.3	32.5±0.3	34.8±0.3	35.8±0.6	36.4±0.4	37.3±0.4	38.2±0.4	39.6±0.4	40.4±0.4	38.3±0.4	40.8±0.4	42.0±0.4	40.1±0.4	41.9±0.4	41.8±0.4
		1-18	30.2 (0.7)	31.7 (0.7)	30.0 (0.7)	34.2 (1.1)	32.8 (0.8)	30.3 (0.7)	30.3 (0.8)	28.2 (0.8)	28.7 (0.8)	29.1 (0.8)	26.1 (0.8)	23.6 (0.8)	26.9 (0.8)	23.2 (0.7)	22.3 (0.7)
		19-64	62.2 (0.7)	60.9 (0.7)	60.4 (0.8)	49.8 (1.2)	51.8 (0.8)	53.6 (0.8)	52.6 (0.8)	53.3 (0.9)	49.8 (0.9)	53.4 (0.9)	51.8 (0.9)	55.1 (0.9)	52.5 (0.9)	56.0 (0.9)	58.3 (0.9)
		≥65	7.6 (0.4)	7.4 (0.4)	9.6 (0.5)	16.0 (0.9)	15.3 (0.6)	16.1 (0.6)	17.2 (0.6)	18.6 (0.7)	21.5 (0.7)	17.4 (0.7)	22.1 (0.8)	21.3 (0.8)	20.7 (0.7)	20.8 (0.7)	19.4 (0.7)
	Residence																
		Urban	64.1 (0.7)	79.1 (0.6)	80.2 (0.6)	71.2 (1.1)	75.2 (0.7)	75.8 (0.7)	79.2 (0.7)	81.1 (0.7)	80.1 (0.7)	79.4 (0.7)	79.2 (0.7)	80.0 (0.7)	80.2 (0.7)	81.3 (0.7)	82.4 (0.7)
		Rural area	35.9 (0.7)	20.9 (0.6)	19.8 (0.6)	28.8 (1.1)	24.8 (0.7)	24.2 (0.7)	20.8 (0.7)	18.9 (0.7)	19.9 (0.7)	20.6 (0.7)	20.8 (0.7)	20.0 (0.7)	19.8 (0.7)	18.7 (0.7)	17.6 (0.7)
	Education																
		≤Elementary school	-	-	36.3 (0.7)	47.4 (1.3)	44.7 (0.8)	41.3 (0.8)	40.5 (0.9)	38.4 (0.9)	39.5 (0.9)	38.5 (0.9)	37.4 (1.0)	35.0 (1.0)	36.1 (0.9)	33.1 (0.9)	30.3 (0.9)
		Middle school	-	-	12.1 (0.5)	10.7 (0.8)	12.9 (0.6)	12.9 (0.5)	12.2 (0.6)	12.4 (0.6)	11.9 (0.6)	11.4 (0.6)	12.8 (0.7)	11.4 (0.6)	10.7 (0.6)	11.4 (0.6)	10.5 (0.6)
		High school	-	-	30.0 (0.7)	23.0 (1.1)	24.0 (0.7)	26.0 (0.7)	24.1 (0.8)	25.5 (0.8)	25.3 (0.8)	26.4 (0.8)	24.9 (0.9)	28.0 (0.9)	23.7 (0.8)	25.0 (0.8)	27.9 (0.8)
		≥College	-	-	21.6 (0.6)	18.9 (1.0)	18.4 (0.7)	19.8 (0.6)	23.2 (0.7)	23.7 (0.8)	23.2 (0.8)	23.7 (0.8)	24.9 (0.9)	25.6 (0.9)	29.5 (0.9)	30.5 (0.9)	31.4 (0.9)
Female (n)			5,416	5,208	4,763	2,270	4,939	5,209	4,469	4,328	4,081	4,046	3,825	3,686	3,977	3,934	3,920
	Age (yr)		35.8±0.3	35.3±0.3	37.3±0.3	40.1±0.5	39.9±0.3	40.6±0.3	40.4±0.3	42.6±0.3	44.0±0.4	41.3±0.4	44.0±0.4	44.8±0.4	42.5±0.4	44.6±0.4	44.9±0.4
		1-18	25.8 (0.6)	26.3 (0.6)	24.2 (0.6)	23.3 (0.9)	23.2 (0.6)	22.1 (0.6)	22.4 (0.6)	20.1 (0.6)	18.2 (0.6)	21.5 (0.6)	18.4 (0.6)	17.0 (0.6)	19.8 (0.6)	17.7 (0.6)	16.8 (0.6)
		19-64	63.2 (0.7)	62.9 (0.7)	63.1 (0.7)	58.2 (1.0)	58.8 (0.7)	59.7 (0.7)	60.2 (0.7)	60.0 (0.7)	59.4 (0.8)	60.2 (0.8)	59.3 (0.8)	60.4 (0.8)	60.1 (0.8)	60.1 (0.8)	60.7 (0.8)
		≥65	11.0 (0.4)	10.8 (0.4)	12.6 (0.5)	18.5 (0.8)	18.1 (0.5)	18.2 (0.5)	17.4 (0.6)	19.9 (0.6)	22.3 (0.7)	18.3 (0.6)	22.4 (0.7)	22.5 (0.7)	20.1 (0.6)	22.2 (0.7)	22.5 (0.7)
	Residence																
		Urban	62.8 (0.7)	78.9 (0.6)	80.8 (0.6)	72.6 (0.9)	76.1 (0.6)	76.2 (0.6)	79.1 (0.6)	81.0 (0.6)	80.9 (0.6)	80.6 (0.6)	81.2 (0.6)	82.0 (0.6)	81.1 (0.6)	82.3 (0.6)	82.4 (0.6)
		Rural area	37.2 (0.7)	21.1 (0.6)	19.2 (0.6)	27.4 (0.9)	23.9 (0.6)	23.8 (0.6)	20.9 (0.6)	19.0 (0.6)	19.1 (0.6)	19.4 (0.6)	18.8 (0.6)	18.0 (0.6)	18.9 (0.6)	17.7 (0.6)	17.6 (0.6)
	Education																
		≤Elementary school	-	-	42.9 (0.7)	48.9 (1.1)	48.6 (0.7)	45.5 (0.7)	45.0 (0.8)	42.8 (0.8)	41.8 (0.8)	40.9 (0.8)	40.9 (0.9)	38.5 (0.9)	38.0 (0.8)	37.7 (0.8)	35.2 (0.8)
		Middle school	-	-	11.5 (0.5)	10.6 (0.7)	10.0 (0.4)	11.3 (0.4)	9.9 (0.5)	11.1 (0.5)	10.5 (0.5)	11.1 (0.5)	10.6 (0.5)	11.7 (0.6)	10.4 (0.5)	10.3 (0.5)	10.0 (0.5)
		High school	-	-	28.8 (0.7)	24.5 (1.0)	25.0 (0.6)	26.0 (0.6)	24.1 (0.7)	25.3 (0.7)	26.0 (0.7)	26.0 (0.7)	25.0 (0.8)	25.4 (0.8)	25.2 (0.7)	23.6 (0.7)	25.7 (0.7)
		≥College	-	-	16.8 (0.5)	16.0 (0.8)	16.4 (0.5)	17.2 (0.5)	21.0 (0.6)	20.8 (0.6)	21.7 (0.7)	22.1 (0.7)	23.4 (0.7)	24.5 (0.8)	26.5 (0.7)	28.4 (0.8)	29.1 (0.8)

Values are presented as mean±stanard error or % (standard error).

**Table 2.** Trends in the major food groups in the Korea National Health and Nutrition Examination Survey from 1998 to 2018^1^

Food groups	1998	2018	Diff	APC	Significant change in the trend slope		
					APC before the year of change	Year of change	APC after the year of change
Grains and cereals							
Total	337.2±3.3	288.4±2.8	-48.8	-0.4^*^	-0.8	2007	-0.2
Male	372.4±4.5	326.8±3.9	-45.6	-0.3	-0.9	2005	0.0
Female	304.0±3.1	248.4±2.8	-55.6	-0.7^*^	-1.0	2007	-0.5
Vegetables							
Total	287.8±4.3	248.1±2.8	-39.7	-0.8^*^	1.4	2005	-1.5^*^
Male	319.7±5.2	283.1±4.0	-36.6	-0.7^*^	1.5	2005	-1.4^*^
Female	257.9±4.1	212.5±3.4	-45.4	-1.0^*^	0.9	2005	-1.6^*^
Fruits							
Total	197.3±5.3	129.2±3.7	-68.1	-0.8	-0.2	2016	-13.3
Male	176.0±5.8	118.4±4.4	-57.6	-0.6	0.0	2016	-13.4
Female	218.5±6.3	140.5±5.0	-78.0	-1.0	-0.5	2016	-12.9
Non-alcoholic beverages							
Total	45.3±2.0	208.4±5.6	163.1	9.2^*^	3.7	2005	10.9^*^
Male	48.7±2.6	226.0±7.9	177.3	9.4^*^	10.1^*^	2016	-0.5
Female	42.1±2.1	189.0±5.9	146.9	8.9^*^	1.9	2005	11.1^*^
Meat							
Total	67.9±1.7	129.8±2.9	61.9	0.5^*^	-0.1	2007	0.8^*^
Male	82.7±2.3	160.0±4.5	77.3	0.7^*^	0.8^*^	2015	-1.1
Female	53.7±1.6	97.9±3.5	44.2	0.1	-0.7	2008	0.6
Milk and dairy products							
Total	79.6±2.6	118.3±3.1	38.7	2.2^*^	3.6^*^	2011	-0.2
Male	79.5±2.9	118.5±3.9	39.0	2.4^*^	3.5^*^	2012	-0.5
Female	79.4±3.0	118.1±4.1	38.7	2.0^*^	3.6^*^	2010	-0.1
Eggs							
Total	21.7±0.6	31.0±1.1	9.3	2.0^*^	1.5	2008	2.4^*^
Male	25.1±0.8	34.4±1.6	9.3	1.7^*^	1.7^*^	2016	-0.5
Female	18.6±0.7	27.6±1.0	9.0	2.2^*^	1.3	2008	2.9^*^

Values are presented as mean±standard error; unit: g/d. Diff, difference between the data from 1998 and 2018; APC, annual percent change.

1 The age-standardized mean and standard error were calculated using the 2005 population projections for Korea.

* p<0.05.

**Table 3.** Energy intake trends in the Korea National Health and Nutrition Examination Survey from 1998 to 2018^1^

Energy intake	1998	2018	Diff	APC	Significant change in the trend slope		
					APC before the year of change	Year of change	APC after the year of change
Energy intake (kcal/d)							
Total	1,934.3±17.9	1,987.7± 17.7	53.4	0.5^*^	-0.1	2007	0.8^*^
Male	2,152.5±22.8	2,301.5±26.1	149.0	0.7^*^	0.8^*^	2015	-1.1
Female	1,729.2±17.1	1,661.1±17.1	-68.1	0.1	-0.7	2008	0.6
Percentage of energy from^2^							
Fat							
Total	17.9±0.2	22.6±0.2	4.7	1.1^*^	0.0	2009	1.9^*^
Male	18.4±0.2	22.7±0.2	4.3	1.0^*^	0.5	2009	1.5^*^
Female	17.4±0.2	22.4±0.2	5.0	1.3^*^	0.2	2009	2.2^*^
Carbohydrates							
Total	67.1±0.2	62.2±0.2	-4.9	-0.3^*^	0.0	2009	-0.6^*^
Male	66.2±0.2	61.6±0.3	-4.6	-0.3^*^	-0.1	2009	-0.6^*^
Female	68.0±0.2	62.8±0.3	-5.2	-0.3^*^	0.0	2009	-0.7^*^
Protein							
Total	15.0±0.1	15.2±0.1	0.2	-0.1	-0.3^*^	2014	1.2
Male	15.4±0.1	15.7±0.1	0.3	-0.1	-0.3^*^	2014	1.4
Female	14.6±0.1	14.8±0.1	0.2	-0.1	-0.3^*^	2014	1.3
Breakfast							
Total	23.1±0.2	16.2±0.3	-6.9	-1.8^*^	-1.4^*^	2009	-2.5^*^
Male	22.5±0.3	16.0±0.3	-6.5	-2.0^*^	-1.3	2005	-2.3^*^
Female	23.6±0.3	16.2±0.3	-7.4	-1.6^*^	-1.0^*^	2009	-2.5^*^
Lunch							
Total	29.6±0.3	29.8±0.3	0.2	-0.2	-0.3^*^	2014	0.8
Male	30.0±0.3	29.0±0.4	-1.0	-0.2	0.2	2005	-0.4
Female	29.4±0.3	30.7±0.4	1.3	-0.1	0.7	2005	-0.4
Dinner							
Total	30.6±0.2	33.7±0.3	3.1	0.2^*^	0.0	2015	3.0^*^
Male	31.8±0.3	35.5±0.4	3.7	0.3^*^	0.1	2015	3.2^*^
Female	29.4±0.3	31.7±0.3	2.3	0.2	-0.3	2012	1.4^*^
Snacks							
Total	16.7±0.4	20.4±0.3	3.7	1.5^*^	2.6^*^	2014	-3.1
Male	15.8±0.4	19.4±0.4	3.6	1.6^*^	2.7^*^	2014	-3.8
Female	17.7±0.5	21.3±0.4	3.6	1.6^*^	2.4^*^	2015	-4.0
Homemade meal							
Total	65.5±0.7	38.4±0.6	-27.1	-2.7^*^	-1.8^*^	2009	-3.8^*^
Male	61.7±0.8	36.5±0.7	-25.2	-2.6^*^	-1.8^*^	2009	-3.7^*^
Female	69.2±0.8	40.4±0.7	-28.8	-2.7^*^	-1.6^*^	2008	-3.8^*^
Dining out meal							
Total	18.9±0.4	36.6±0.6	17.7	2.4^*^	1.4	2011	4.0^*^
Male	23.1±0.5	39.7±0.8	16.6	2.0^*^	1.4	2012	3.5
Female	15.0±0.5	33.3±0.7	18.3	2.5^*^	0.9	2011	5.1^*^
Convenience food							
Total	15.5±0.5	25.1±0.4	9.6	3.2^*^	5.3^*^	2012	0.1
Male	15.2±0.5	23.9±0.5	8.7	3.1^*^	4.4^*^	2012	0.8
Female	15.8±0.6	26.3±0.5	10.5	3.1^*^	5.1^*^	2013	-1.2

Values are presented as mean±standard error. Diff, difference between the data from 1998 and 2018; APC, annual percent change.

1 The age-standardized mean and standard error were calculated using the 2005 population projections for Korea.

2 The percentage of energy from fat means the percentage of energy from fat (g of fat×9 kcal/g) compared to the sum of energy from fat, carbohydrates, and protein; The respective percentages of energy from the other components were calculated using a similar equation.

* p<0.05.

**Table 4.** Nutrient intake trends in the Korea National Health and Nutrition Examination Survey from 1998 to 2008^1^

Nutrient	Sex	1998	2018	Diff	APC	Significant change in the trend slope		
						APC before the year of change	Year of change	APC after the year of change
Vitamin A (μgRE/d)	Total	610.1±11.9	571.7±9.7	-38.4	-0.5	2.4^*^	2011	-5.3^*^
	Male	671.9±14.3	621.5±14.0	-50.4	-0.5	2.4^*^	2011	-5.6^*^
	Female	552.1±13.0	520.7±11.4	-31.4	-0.3	1.9^*^	2012	-5.9^*^
Riboflavin (mg/d)	Total	1.08±0.01	1.64±0.02	0.56	2.0^*^	0.6	2008	3.4^*^
	Male	1.19±0.02	1.86±0.03	0.67	2.3^*^	0.4	2007	3.4^*^
	Female	0.97±0.01	1.41±0.02	0.44	1.7^*^	0.0	2008	3.4^*^
Vitamin C (mg/d)	Total	123.8±2.3	60.6±1.4	-63.2	-3.2^*^	-1.9^*^	2014	-11.7^*^
	Male	122.3±2.5	65.3±2.2	-57.0	-3.0^*^	-1.3^*^	2012	-8.8^*^
	Female	125.5±2.7	55.6±1.5	-69.9	-3.4^*^	-2.3^*^	2015	-15.3^*^
Calcium (mg/d)	Total	500.7±7.5	516.2±6.3	15.5	0.1	-0.1	2014	1.0
	Male	541.4±9.5	571.6±8.1	30.2	0.2	0.1	2014	0.9
	Female	462.0±7.1	459.2±7.4	-2.8	-0.1	-0.3	2015	1.7
Sodium (mg/d)	Total	4,585.6±62.4	3,255.0±37.4	-1,330.6	-2.3^*^	-0.3	2010	-4.9^*^
	Male	5,130.5±73.4	3,809.5±51.7	-1,321.0	-1.9^*^	0.3	2010	-5.0^*^
	Female	4,068.7±63.1	2,679.6±37.9	-1,389.1	-2.8^*^	1.4	2005	-4.1^*^
Iron (mg/d)	Total	12.5±0.2	11.4±0.1	-1.1	-0.4	1.7^*^	2014	-8.1^*^
	Male	13.7±0.2	13.0±0.2	-0.7	0.2	1.5^*^	2015	-10.9^*^
	Female	11.3±0.2	9.7±0.1	-1.6	-0.5	1.7^*^	2014	-9.2^*^

Values are presented as mean±standard error. Diff, difference between the data from 1998 and 2018; APC, annual percent change.

1 The age-standardized mean and standard error were calculated using the 2005 population projections for Korea.

* p<0.05.

## SUPPLEMENTARY MATERIALS

Korean version is available at http://www.e-epih.org/.
