# Supplementary material for: Trends in food and nutrient intake over 20 years: findings from the 1998-2018 Korea National Health and Nutrition Examination Survey
Source: Epidemiol Health. 2021 Apr 19;43:e2021027. doi: 10.4178/epih.e2021027 (PMC8289469; doi:10.4178/epih.e2021027)
Supplement: Supplementary file 1 [file epih-43-e2021027-suppl.docx]

**Title:**

**국민건강영양조사 20년(1998-2018): 식품 및 영양소 섭취 추이**

Trends in food and nutrient intake over 20 years: findings from the 1998-2018 Korea National Health and Nutrition Examination Survey

**Running title**: Twenty-year trends in food and nutrient intake

**Authors:**

권상희^1^, 박진영^1^, 박명숙^1^, 김양하^1^, 연소영^1^, 윤이나^1^, 윤성하^1^, 박수연^1^, 양지은^1^, 김영택^2^, 박옥^1^, 오경원^1^

Sanghui Kweon^1*^, Jin Young Park^1^, Myungsook Park^1^, Yangha Kim^1^, So Yeong Yeon^1^, Leena Yoon^1^, Sungha Yun^1^, Suyeon Park^1^, Ji Eun Yang^1^, Youngtaek Kim ^2^, Ok Park^1^, Kyungwon Oh^1^

**Affiliations:**

^1^질병관리청 만성질환관리국 건강영양조사분석과; ^2^충남대학교 병원

^1^Division of Health and Nutrition Survey and Analysis, Bureau of Chronic Disease Prevention and Control, Korea Disease Control and Prevention Agency, Cheongju, Korea; ^2^Public Health Medical Service Office, Chungnam National University Hospital, Daejeon, Korea

**Correspondence:** Kyungwon Oh

Division of Health and Nutrition Survey and Analysis, Bureau of Chronic Disease Prevention and Control, Korea Disease Control and Prevention Agency, 187 Osongsaengmyeong 2-ro, Heungduk-gu, Cheongju 28159, Korea

E-mail: [kwoh27@korea.kr](mailto:kwoh27@korea.kr)

**ABSTRACT**

**목적**: 국민건강영양조사 자료를 활용하여 우리 국민의 20년간의 식품 및 영양소 섭취 현황 및 추이를 파악하고자 하였다.

**연구방법**: 조사대상은 국민건강영양조사 제1기(1998)~제7기(2016-2018) 영양조사(식품 섭취조사)에 참여한 만1세이상 116,284명이었다. 식품섭취조사는 24시간회상법을 이용하여 조사일 하루 전날 섭취한 내용을 조사한 자료이며, 식품군 및 영양소 섭취량의 연간 변화율(Annual Percent Change, APC)은 SAS 및 Joinpoint 프로그램을 이용하여 산출하였다.

**결과**: 곡류와 채소류 섭취는 20년간 각각 50g(1998년 337g, 2018년 288g(APC=-0.4, p<0.05), 40g(1998년 288g, 2018년 248g, APC=-0.8, p<0.05) 정도 감소하였다. 반면 음료류, 육류, 우유류, 난류 섭취량은 증가했으며 특히 음료류 섭취가 1998년 45g에서 2018년 208g으로 4배 이상 증가하였다(APC=9.2, p<0.05). 에너지 섭취량은 큰 변화가 없었으나, 탄수화물에 의한 에너지 섭취분율은 약 5%p 감소(1998년 67.1%, 2018년 62.2%, APC=-0.3, p<0.05), 지방분율은 약 5%p 증가(1998년 17.9%, 2018년 22.6%, APC=1.1, p<0.05)했다. 또한, 아침식사, 가정식 섭취분율이 감소하고 간식, 외식, 식품 및 편이식품 분율이 증가하는 등 에너지 섭취량의 구성요소 간 변화가 뚜렷하였다. 영양소에서는 비타민 C(1998년 124mg, 2018년 61mg, APC=-3.2, p<0.05)와 나트륨 섭취(1998년 4,586mg, 2018년 3,255mg, APC=-2.3, p<0.05)가 큰 폭으로 감소하였다.

**결론**: 지난 20년 간 곡류, 채소류 감소, 음료류, 우유류, 육류, 난류 증가 등 식품 섭취 변화가 있었고, 지방 섭취 증가, 탄수화물, 나트륨, 비타민 C 감소 등 영양소 섭취에도 변화가 있었다. 영양상태는 만성질환 발생과 관련한 중요한 요인이므로 섭취수준에 대한 지속적인 평가 및 섭취량 변화에 대한 감시가 필요하다.

**주제어**: 국민건강영양조사, 식품군 섭취, 영양소 섭취

**INTRODUCTION**

질병부담 연구(Global Burden of Disease Study)에서는 식생활 개선을 통해 5명 중 1명의 사망을 예방할 수 있다고 보고하고 있다. 그러나 전세계적으로 만성질환 예방에 바람직한 식품(견과류, 우유, 전곡류 등)의 섭취는 적정기준에 비해 적게 섭취하는 반면 나트륨, 가당음료, 가공육 등 섭취를 권고하지 않는 식품은 기준보다 많이 섭취하고 있어 만성질환으로 인한 사망 및 질병부담의 감소를 위해 개선방안 마련이 필요함을 제언하였다 [1]. 우리나라의 경우 영양요인이 총 사망 기여 위험요인 중 흡연, 고혈당에 이어 세 번째를 차지하고 있으며, 나트륨 과잉 섭취, 섬유소 및 통곡물 섭취 부족이 주요한 사망 기여 위험요인이었다 [2].

이처럼 영양은 심뇌혈관질환, 암, 당뇨병 등 만성질환의 예방 및 관리에 있어서 중요한 요인으로 우리나라를 비롯한 대부분의 국가에서 만성질환 예방 및 관리 중재 방안 마련을 위해 국가 단위 조사를 수행하여 식품 및 영양 섭취수준 변화를 파악하고 있다. 미국의 경우 National Health and Nutrition Examination Survey를 실시하여 연령, 인종별 영양섭취, 식사의 질 등 변화를 파악하여 건강정책의 근거로 활용하고 있고 [3], 일본은 National Health and Nutrition Survey에 기반하여 영양 섭취수준을 지속적으로 평가하고 있다[4]. 우리나라는 국민건강영양조사를 통해 우리 국민의 영양상태 평가, 영양취약집단 선별에 활용하고, 제4차(2020) 국민건강증진종합계획(Health Plan 2020, HP2020) 목표치 대비 비교 등 영양 정책의 성과를 평가하고 있다 [5].

우리나라는 암, 심뇌혈관질환, 당뇨병 등 만성질환에 의한 사망이 전체 사망의 약 80%를 차지하고 있으며 [6], 지난 20년간 비만 및 고콜레스테롤혈증 유병률은 증가, 고혈압 및 당뇨 유병률은 정체되는 등 주요 만성질환 유병률은 악화되고 있는 실정이다 [7]. 따라서 만성질환의 주요 위험요인인 영양 섭취수준 변화에 대한 심층적 파악을 통해 예방 및 관리 방안을 마련할 필요가 있다. 질병관리청에서 매년 국민건강통계를 발표하고 있지만 검진조사, 건강설문조사, 영양조사 결과를 모두 포함하고 있어 영양섭취에 대한 심층적인 결과 해석이나 고찰은 부족하다. 일부 선행연구[8, 9]에서 동물성 식품군과 지방 섭취 증가 등 우리 국민의 식생활 변화 추이에 대해 보고하였지만 1998년 이전의 과거 자료에 대한 분석 결과로 최신의 식생활 추이 변화에 대한 파악이 필요하며, 최근의 자료를 이용하여 분석한 선행연구[10, 11]의 경우 에너지, 나트륨 등 일부 영양소 위주로 결과를 제시하고 있어 전반적인 식생활의 변화를 파악하기에는 제한적이다.

이에 본 논문에서는 국민건강영양조사(1998년~2018년) 자료를 분석하여 주요 식품 및 영양소 섭취의 20년간 변화를 파악하여 만성질환 예방 및 관리에 필요한 근거를 제공하고자 한다.

**SUBJECTS AND METHODS**

**조사대상**

국민건강영양조사는 1995년에 제정된 국민건강증진법에 근거하여 1998년부터 국가 단위 건강통계 생산을 위해 실시하고 있다. 국민건강영양조사는 1998년과 2001년에는 11-12월, 2005년에는 4-6월(영양조사 4-5월), 2007년에는 7-12월에 실시되었고, 2008년부터 계절적 편향 없이 매년 통계 생산이 가능하도록 연중조사(1-12월)로 수행되고 있다. 조사 표본은 1998년부터 조사구 및 가구를 1, 2차 추출단위로 2단계 층화집락표본추출방법을 동일하게 적용하여 추출하였다. 기본 추출틀은 인구주택총조사 자료이며, 층화변수는 시도, 동읍면, 주택유형(일반/아파트)을 적용하였다. 2010년이후부터는 표본설계 시 내재적 층화변수로 주거면적, 가구주 학력 등을 추가로 고려하여 변화된 모집단 특성을 반영함과 동시에 표본의 대표성과 시계열적 일관성을 유지한 통계 생산이 가능하도록 하였다.

조사대상은 위의 방법을 이용하여 추출된 표본가구 내 만1세 이상의 모든 가구원이었다. 본 연구의 분석 대상은 국민건강영양조사 제1기(1998)부터 제7기(2016-2018) 영양조사의 식품섭취조사를 완료한 만1세이상 총 116,284명이었다.

**영양조사**

이 중 식품섭취조사는 24시간회상법으로 조사일 하루 전날 섭취한 모든 음식에 대해 시간 순서대로 식사 장소, 매식 종류 등과 함께 음식 종류와 섭취량을 수집하는 조사이며, 영양사로 구성된 조사팀이 대상자 가구를 방문하여 만 1세 이상 대상에게 개인별 면접을 통해 수행되었다 [12]. 정확한 섭취량 파악을 위해 2차원 모델집, 계량컵, 계량스푼, 자 등의 보조도구를 이용하여 개인별 섭취량을 조사하였고, 가구 내에서 조리된 음식을 섭취한 경우 조리자를 대상으로 음식별로 사용된 재료 식품의 양과 음식의 총량을 조사하여 대상자의 식품 및 영양소 섭취량 산출 시 반영하였다. 조리 조사가 불가할 경우에는 음식별 식품재료량 데이터베이스 (Database, DB)를 활용하였다. 개인별로 1일 간 섭취한 내용은 국가표준식품성분표를 기반으로 구축한 식품별 영양성분 DB를 활용하여 최종적으로 에너지 및 영양소 섭취량을 산출하였다 [7].

본 연구에서 식품군은 연도별 섭취량 차이가 있는 곡류, 채소류, 음료류, 과일류, 육류, 우유류, 난류를, 영양소는 에너지와 탄수화물, 단백질, 지방으로부터 섭취한 에너지분율과 HP2020 영양과제 지표의 구성요소인 비타민 A, 리보플라빈, 비타민 C, 칼슘, 나트륨, 철을 중점적으로 검토하였다. 에너지의 급원 영양소인 지방, 탄수화물, 단백질은 에너지 구성의 변화를 검토하기 위해 에너지섭취분율로 제시하였다. 국민건강영양조사에서 비타민 A 섭취량은 2015년까지 ㎍ RE를 단위로 발표되었으나, 2015 한국인 영양소 섭취기준의 비타민 A에 대한 기준 단위가 변경됨에 따라 제7기(2016-2018) 조사부터는 ㎍ RAE로 발표되고 있다. 본 연구에서는 20년간의 비교가 가능하도록 비타민 A의 단위를 ㎍ RE로 통일하여 섭취량을 비교하였다. 식품군 섭취량과 에너지 및 영양소 섭취량 외에도 식생활 변화 파악을 위해 식사 끼니별 에너지 섭취 분율과 가정식, 외식, 편이식품 등과 같은 식사 종류별 에너지 섭취 분율을 추가로 검토하였다. 식사 끼니는 아침식사, 점심식사, 저녁식사와 간식으로 구분하였고, 식사 종류는 가정식, 외식(음식업소 음식 및 단체급식), 편이식품(즉석조리식품, 반조리식품 등) 및 식품(우유, 과일, 과자 등)으로 구분하였다.

**분석방법**

모든 결과는 목표 모집단인 우리나라에 거주하는 국민의 특성을 잘 대표할 수 있도록 가중치를 적용한 복합표본설계분석방법으로 산출하였다. 식품군 및 영양소에 따른 연도별 섭취량(또는 분율)은 연령 구조 차이에 따른 영향을 보정하기 위해 2005년 추계인구로 표준화하여 제시하였다. 자료의 분석은 SAS version 9.4 (SAS Institute Inc., Cary, NC, USA)와 Joinpoint Regression Program version 4.1.1.1 (US National Cancer Institute, Bethesda, MD, USA)을 이용하였다. 식품군 및 영양소 섭취량(또는 분율)의 연간변화율(Annual Percent Change, APC)은 표준화된 평균과 표준오차를 Joinpoint software에 적용하여 변곡점(joinpoint)을 0, 1개로 설정, 모형을 추정하여 산출하였다. APC는 유의수준 0.05하에서 연간변화율이 “0”인지 검증하였고, 변곡점(joinpoint) 수에 대한 최적모형검정은 Joinpoint Regression Program의 Monte Carlo method를 사용하였다.

**윤리 성명**

본 연구는 질병관리본부 연구윤리심의위원회를 통해서 연도별 계획에 따라 승인을 받았으며(2007-2014년, 2018년), 일부 연도(2015-2017년)에는 생명윤리법 제2조제1호 및 동 시행규칙 제2조제2항제1호에 따라 심의면제를 받았다.

**RESULTS**

본 연구대상은 국민건강영양조사(1998-2018) 영양조사 중 식품섭취조사를 완료한 만1세이상 총 116,284명(남자 52,213명, 여자 64,071명)이었다. 20년간 평균 연령이 1998년 34.5에서 2018년 43.5세로 약 10세 증가하였고 교육수준별로는 대졸이상 비율이 약 10%p 증가하였다 (Table 1).

국민건강영양조사가 도입된 1998년 이후 곡류 섭취량은 감소하는 경향이었으며(APC=-0.4, p<0.05), 특히 여자 섭취량의 감소가 유의하게 나타났다(Table 2). 채소류 섭취량은 2005년 이후 감소했으며(APC=-1.5, p<0.05), 과일류는 전반적으로 감소 경향을 보였으나 통계적으로는 유의하지 않았다. 지난 20년간 섭취량 변화가 가장 큰 식품군은 음료류로, 2005년 이후 섭취량이 크게 증가하여 1998년 당시 수준에 비해 4.6배가 되었다(1998년 45.3g, 2018년 208.4g, APC=9.2, p<0.05). 동 기간 동안 육류 섭취량도 증가하였으며 특히 남자 섭취량은 지난 20년간 유의하게 증가하여 약 2배가 되었다(1998년 82.7g, 2018년 160.0g, APC=0.7, p<0.05). 우유류 섭취량은 2011년까지 지속적으로 증가하는 경향이었으나(APC=3.6, p<0.05) 이후에는 큰 변화가 없었고, 난류는 전반적으로 증가하여 2018년 섭취량이 31.0g으로 1998년 21.7g의 1.5배 수준이었다(APC=2.0, p<0.05).

에너지 섭취량은 지난 20년간 전체적으로 증가하는 경향이었으나 차이는 53kcal로 크지 않았다(APC=0.5, p<0.05). 남자의 섭취량은 유의하게 증가한 반면 여자는 20년동안 유의한 변화가 없었다(Table 3). 지방으로 섭취한 에너지 분율은 1998년 17.9%에서 2018년 22.6%로 유의하게 증가했고(APC=1.1, p<0.05), 2009년 이후 남녀 모두 증가가 가속화되었다. 같은 기간 탄수화물로부터 섭취한 에너지 분율은 4.9%p(1998년 67.1%, 2018년 62.2%) 감소하였으며 단백질 에너지섭취분율은 큰 변화가 없었다. 끼니나 매식 여부 등의 경우 아침식사를 통해서 섭취하는 에너지 분율은 크게 감소한 반면(1998년 23.1%, 2018년 16.2%, APC=-1.8, p<0.05) 간식으로 섭취하는 분량이 상대적으로 증가하였다(APC=1.5, p<0.05). 에너지 섭취량 중 가정식으로부터 섭취한 분율은 감소한 반면 외식 분율은 약 2배(1998년 18.9%, 2018년 36.6%), 식품 또는 편이식품으로 섭취하는 에너지 분율도 약 1.5배(1998년 15.5%, 2018년 25.1%) 증가하였다.

영양소의 경우 비타민 C, 나트륨의 섭취는 유의하게 감소하였고 리보플라빈의 섭취는 유의하게 증가하였다(Table 4). 비타민 C 섭취 감소 경향은 2014년을 기점으로 가속화되었다(APC=-11.7, p<0.05). 나트륨 섭취량은 1998년 4,585.6 mg에서 2018년 3,255.0 mg으로 20년간 크게 감소했으며, 특히 2010년 이후 가파르게 감소했다(APC=-4.9, p<0.05). 비타민 A 섭취는 2011년까지 증가 경향이었으나(APC=2.4, p<0.05) 이후 감소하는 경향이었다(APC=-5.3, p<0.05). 칼슘 섭취량은 변화가 없었으며 철 섭취량은 2014년 이후 감소 경향이었다(APC=-8.1, p<0.05).

**DISCUSSION**

국민건강영양조사가 도입된 1998년 이후 지난 20년간 곡류, 채소, 과일류 섭취량은 감소하고 음료류, 육류, 우유류, 난류 섭취량은 증가했다. 이러한 식품군의 변화는 영양소 섭취량 변화와도 관련되어 비타민 C 섭취는 감소하고, 리보플라빈 섭취가 증가하였다. 총 에너지 섭취량은 남자에서만 다소 증가하는 경향을 보였으나 그 변화 폭이 크지는 않았다. 다만, 이를 구성하는 요소에는 여러 가지 변화가 있었는데 지방 섭취로 인한 에너지 분율이 증가하였고 외식과 식품 및 편이식품으로 섭취하는 에너지 분율이 증가했다.

본 연구에서 식물성 식품의 섭취량은 감소, 동물성 식품의 섭취량은 증가하였고, 동시에 탄수화물로 섭취하는 에너지 분율은 감소, 지방의 에너지 섭취 분율은 증가하였다. 동물성 식품 및 지방 섭취 증가는 1969년에서 1995년의 식품섭취 변화를 분석한 연구 등 국내 선행연구에서 보고된 결과와도 유사하여 1998년 이전부터 변화 경향이 있었음을 확인할 수 있었다 [8, 9]. 우리나라는 1인가구 증가와 같은 가구유형 변화 [13], 여자의 경제활동참여 증가 [14] 등 사회경제적 환경이 변화하고 있고, 식습관도 이에 영향을 받아 변화하고 있다. 본 연구에서 식사끼니별, 식사 종류별 에너지 섭취분율 추이에 대해 검토한 결과, 아침식사와 가정식의 에너지 섭취분율은 감소하고, 간식, 외식, 식품 및 편이식품의 에너지 섭취분율은 증가하는 등 식습관의 변화를 확인할 수 있었다. 이러한 식습관의 변화는 식품군 섭취량 변화에 일부 기여했을 것으로 판단된다.

지난 20년 동안 중량 기준으로 섭취량이 가장 크게 변한 식품군은 음료류로, 남녀 각각 변곡점이 발생한 연도가 다르긴 하지만 모두 섭취량이 유의하게 증가하였다. 당이 첨가된 음료류(가당음료)는 에너지 및 첨가당 과잉 섭취에 대한 우려로 가급적 섭취를 줄이고자 노력해야 하는 식품군이다 [15, 16]. 우리나라 만1세 이상의 음료류를 통한 에너지 섭취량은 1998년 30.7kcal에서 2018년 72.5kcal로 41.8kcal가 증가하여 음료류 섭취량의 증가가 총 에너지 섭취량 증가에 영향을 주었을 것으로 보인다. 또한 당이 첨가된 커피, 탄산음료, 과일음료 등의 음료류가 당 섭취량 주요 급원식품으로 산출된 점에서 음료류 섭취가 당 섭취량에 기여했을 것으로 판단된다 [7]. 향후 가당음료를 세분화한 음료류 섭취량 추이 분석 및 식품별 첨가당 DB 등 관련 DB 구축을 통한 추가 연구가 필요할 것으로 보인다.

채소류의 경우 음료류와는 반대로 20년간 섭취가 유의한 수준으로 감소하였고, 과일류 섭취량은 감소 경향을 보였지만 통계적으로 유의하지는 않았다. 채소와 과일은 다른 식품군보다 계절에 따라 섭취량이 차이가 있을 수 있다는 점을 고려하여 연중조사 도입 이후인 2008-2018년 자료로 연간변화율을 분석한 결과, 2015년 이후로 과일 섭취량은 매년 남, 여 모두 유의하게 감소하였고, 채소류 섭취도 2014년부터 감소폭이 더 큰 것을 확인할 수 있었다 (data not shown). 채소 및 과일류는 식이섬유를 비롯하여 각종 비타민, 무기질 섭취에 중요한 급원이며 만성질환 예방 및 관리를 위해 권고하는 식품인 점을 고려하여 섭취 증가를 위한 정책적 노력이 필요할 것으로 보인다. 채소 및 과일류 섭취가 500g 이상인 19세 이상 성인 비율은 2018년 29.4%로 1998년 42.9%에 비해 13.5%p 감소하였는데, 특히 19-29세(25.1%p 감소), 30-49세(19.7%p 감소)에서 큰 폭으로 감소하여 청장년층의 섭취량 감소에 대한 원인 분석이 필요할 것으로 사료된다 [7].

에너지 섭취량은 2018년 기준(1세이상) 1,988kcal로 20년간 소폭(53kcal) 증가하였고 특히 남자(149kcal)에서 유의한 수준으로 증가하였다. 여자의 에너지 섭취량은 20년간 비교 시 유의한 추이 변화가 없었으나 2008-2018년 10년간 연간변화율은 증가하는 경향이었다 (APC=0.7, p<0.05; data not shown). 미국의 에너지 섭취량은 2,093kcal (2017∼2018년, 2세이상)이었고 [3], 일본(2018년, 1세이상)의 에너지 섭취량은 1,900kcal으로 우리나라와 비교 시 큰 차이는 없었으나 [4] 우리나라와 달리 20년간(1995-2016년) 일본의 에너지 섭취량은 감소하는 경향이었다 [17].

에너지 섭취량의 변화는 크게 없었지만 남, 녀 모두 에너지 섭취량 중 탄수화물의 기여 분율은 감소했고, 지방의 기여 분율은 2018년 기준(1세이상) 22.6%(지방 섭취량 49.5g)으로 지난 20년간 4.7%p 증가했으며 이는 남, 녀 모두 통계적으로도 유의하였다. 이러한 지방 에너지섭취분율의 증가 경향은 미국과 일본의 연구에서도 보고되었다 [17, 18]. 우리나라의 지방 에너지섭취분율은 미국 36.0%(지방섭취량 85.0g), 일본 28.3%(섭취량 60.4g) 보다 낮은 수준이며 2015 한국인 영양소 섭취기준과 비교해도 적정 범위 이내였다. 그러나 20년 동안 약 5%p, 특히 최근 10년 동안 4%p 증가하였으며, 총 에너지섭취량 중 지방으로 섭취하는 에너지가 30%를 초과하는 사람의 비율도 증가했는데 특히 젊은 연령층인 20대(1998년 14%, 2018년 29%)와 30대 (1998년 9%, 2018년 24%)가 큰 폭으로 증가했다 (data not shown). 이러한 경향과 함께 고콜레스테롤혈증과 비만 유병률 증가 추세 등을 고려하면 지방 섭취량 및 지방 에너지섭취분율에 대한 지속적인 감시가 필요하다고 할 수 있다.

영양소 섭취량은 식품군 섭취량의 영향을 받아 변화가 있었다. 채소류와 과일류 섭취량의 감소는 비타민 C의 감소에, 육류, 우유류, 난류 섭취량의 증가는 리보플라빈 섭취량의 증가에 기여했을 것으로 보인다. 반면 칼슘 섭취량의 주요 급원식품인 우유류 섭취는 증가했으나 채소류 섭취가 감소하여 칼슘 섭취량은 지난 20년간 변화 없이 비슷한 수준이었다. 가장 섭취량의 변화가 큰 영양소는 나트륨으로, 나트륨 섭취량은 남, 녀 모두 통계적으로 유의하게 감소하였다. 나트륨 섭취량의 감소는 식생활의 변화로 인한 주요 급원식품 섭취량 감소, 나트륨 저감화 정책 등 여러 요소가 복합적으로 작용했을 것으로 판단된다. 나트륨의 주요 급원식품인 배추김치의 섭취량은 1998년 83.8g에서 2018년 62.9g으로 감소하였다 [7, 19]. 또한 우리나라는 나트륨 저감화 필요성이 대두되면서 2007년 어린이 급식 나트륨 저감화를 시작으로 2012년부터 국가에서 유탕면류, 장류, 과자 등 가공식품 및 단체급식의 나트륨 함량 저감화 사업 추진과 국민 인식을 개선하기 위한 정책을 시행하였다 [20]. 이와 같은 이유로 인해 나트륨 섭취량은 2018년 3,255mg으로 1998년 4,586mg에 비해 크게 감소하였으나 여전히 권고 기준인 2,000mg 보다 높고, 만9세 이상의 약 75%가 2,000mg 이상을 섭취하고 있어 나트륨 섭취 감소를 위한 정책의 지속 추진이 필요하다 [7].

국민건강영양조사 식품섭취조사는 20년간 같은 방법(24시간회상법)으로 실시하였으나 연도별 조사 시기, 조사 결과 산출 시 사용한 음식별 식품재료량 DB, 식품별 영양성분 DB에 차이가 있으며, 이는 이 연구의 제한점이다. 우선, 결과에서 기술한 바와 같이 2008년 이전에는 조사연도별로 조사시기가 달라 계절에 따른 차이가 있는 식품 및 영양소의 경우 이를 고려한 해석이 필요하다. 예를 들어 과일류 섭취량은 1998년과 2001년(조사시기 11-12월) 각각 197.3g, 208.3g, 2005년(조사시기 4-5월)에는 87.6g으로 평균 섭취량에 100g 이상 차이가 있었고 [7], 이는 조사 시기의 영향을 받았을 가능성을 배제할 수 없다. 이에 연중조사가 정착된 2008년을 기준으로 2018년까지의 식품 및 영양소의 섭취 추이를 추가로 분석하였다. 과일류의 경우 20년간의 추이는 유의한 수준의 변화가 없었으나 2008년-2018년 추이 분석 시 2015년 이후 감소 경향이었다(APC=-11.1, p<0.05). 20년간 유의한 수준의 변화가 없었던 여자 에너지 섭취(APC=0.7, p<0.05), 단백질 에너지섭취분율(APC=0.4, p<0.05), 비타민 A 섭취(APC=-3.4, p<0.05)도 10년간 추이에는 유의한 변화 경향이 있었다. 이를 제외한 식품 및 영양소는 연간 변화율 크기에 다소 차이가 있었지만 증가 또는 감소 경향은 20년간의 추이와 차이가 없었다 (data not shown). 다음으로 국민건강영양조사는 당해년도 영양상태 현황 파악이 주요한 목적이므로 결과 산출 시 가용한 최신의 음식별 식품재료량 DB, 식품별 영양성분 DB를 적용하고 있다. 최신 DB로의 변경은 영양상태를 평가하는 시점의 음식 정보 또는 식품의 영양성분 정보를 반영할 수 있다는 장점이 있지만, 식품 및 영양소 섭취량 추이 평가 시에는 적용된 DB의 영향을 고려해야 하는 제한점이 있다. 그 예로 철 섭취량의 경우 국가표준식품성분표 제9.1개정판[21]을 사용한 결과가 제8개정판[22]을 사용한 결과에 비해 낮게 산출되었으므로(data not shown), 제8개정판이 적용된 제6기(2013-2015) 국민건강영양조사 결과와 제9.1개정판이 적용된 제7기(2016-2018) 결과를 비교할 때에는 적용된 DB 차이를 함께 고려해야 한다. 자료처리용 DB 변경 시에는 동일한 원자료에 대해 기존 DB와 변경할 DB를 각각 적용하여 산출한 결과를 관계 기관 및 전문가 검토를 거쳐 확인하고 있으며, 결과 비교 시 참조할 수 있도록 국민건강영양조사 통계집, 원시자료 이용지침서에 조사 결과 산출 시 사용한 DB 정보를 상세하게 기술하여 공개하고 있다.

지난 20년간의 국민건강영양조사 결과에서, 나트륨 섭취 감소를 제외하면 채소 및 과일 섭취가 감소하고 음료류와 지방 섭취가 증가하는 등 긍정적인 변화는 드물었다. 식품 및 영양소 섭취는 만성질환 예방의 주요한 요인이므로 이러한 추이를 개선하기 위한 영양정책 개발과 적극적인 추진이 필요하다. 또한, 국민건강영양조사는 시의적 정책 개발을 지원하기 위한 사업이므로 영양 문제 점검, 만성질환 관련 영양요인을 발굴할 수 있도록 조사방법 개선과 다양한 분석 시도가 필요하다.

**CONFLICT OF INTEREST**

모든 저자는 본 연구에 대해 표명할 이해상충이 없음

**FUNDING**

없음

**ACKNOWLEDGEMENTS**

지난 20년동안 국민건강영양조사에 참여해주신 대상자께 감사합니다. 또한, 조사를 전담하여 수행한 조사수행팀, 조사 지원과 자문을 주신 관련학회 및 전문가 자문단에게도 감사드립니다.

**AUTHOR CONTRIBUTIONS**

Conceptualization: SK. Data curation: SP, JEY. Formal analysis: JYP, MP, SYY, LY, SY, YK. Funding acquisition: None. Methodology: YK, OP, KO. Project administration: SK, MP. Writing - original draft: SK, SP, JEY, KO. Writing - review and editing: JYP, MP, YK, SYY, LY, SY, YK, OP, KO.

**ORCID**

Sanghui Kweon: *https://orcid.org/0000-0002-2678-8858*; Jin Young Park: [*https://orcid.org/0000-0001-7157-3875*](https://orcid.org/0000-0001-7157-3875); Myungsook Park: [*https://orcid.org/0000-0002-0725-5677*](https://orcid.org/0000-0002-0725-5677); Yangha Kim: [*https://orcid.org/0000-0002-5895-3790*](https://orcid.org/0000-0002-5895-3790); So Yeong Yeon: [*https://orcid.org/0000-0003-2505-4411*](https://orcid.org/0000-0003-2505-4411); Leena Yoon: *https://orcid.org/ 0000-0002-2200-17 98*; Sungha Yun: [*https://orcid.org/0000-0002-3624-4512*](https://orcid.org/0000-0002-3624-4512); Suyeon Park: *https://orcid. org/0000-0001-8134-8436*; Ji Eun Yang: [*https://orcid.org/0000-0002-6245-613X*](https://orcid.org/0000-0002-6245-613X); Youngtaek Kim: *https:// orcid.org/0000-0003-0139-7620*; Ok Park: [*https://orcid.org/0000-0002-9477-9523*](https://orcid.org/0000-0002-9477-9523); Kyungwon Oh: *https:// orcid.org/0000-0001-8097-6078.*

**REFERENCES**

1. GBD 2017 Diet Collaborators. Health effects of dietary risks in 195 counties, 1990-2017: a systematic analysis for the Global Burden of Disease Study 2017. Lancet 2019;393:1958-1972.
2. Institute for Health Metrics and Evaluation. GBD compare: Republic of Korea [cited 2020 Nov 19]. Available from: https://vizhub.healthdata.org/gbd-compare/.
3. U.S. Department of Agriculture. What we eat in America, NHANES 2017-2018 [cited 2020 Sep 19]. Available from https://www.ars.usda.gov/northeast-area/beltsville-md-bhnrc/beltsville-human-nutrition-research-center/food-surveys-research-group/docs/wweia-data-tables/.
4. National Institute of Health and Nutrition of Japan. National Health and Nutrition Survey [cited 2020 Sep 19]. Available from https://www.nibiohn.go.jp/eiken/kenkounippon21/en/eiyouchousa/

kekka_eiyou_chousa.html (Japanese).

1. Kweon S, Kim Y, Jang MJ, Kim Y, Kim K, Choi S, et al. Data resource profile: the Korea National Health and Nutrition Examination Survey (KNHANES). Int J Epidemiol 2014;43:69–77.
2. Korea Centers for Disease Control and Prevention. Factbook: Non-communicable Diesease 2019. Cheongju: Korea Centers for Disease Control and Prevention; 2020, p. 6 (Korean).
3. Korea Centers for Disease Control and Prevention. Korea Health Statistics 2018: Korea National Health and Nutrition Examination Survey (KNHANES VII-3) [cited 2020 Sep 19]. Available from https://knhanes.kdca.go.kr/knhanes/sub04/sub04_04_01.do (Korean).
4. Kim S, Moon S and Popkin BM. The nutrition transition in South Korea. Am J Clin Nutr 2000;71:44-53.
5. Lee SK and Sobal J. Socio-economic, dietary, activity, nutrition and body weight transitions in South Korea. Public Health Nutr 2003;6:665-674.
6. Yun S, Kim HJ and Oh K. Trends in energy intake among Korean adults, 1998-2015: results from the Korea National Health and Nutrition Examination Survey. Nutr Res Pract 2017;11:147-154.
7. Park CY, Jo G, Lee J, Singh GM, Lee JT and Shin MJ. Association between dietary sodium intake and disease burden and mortality in Koreans between 1998 and 2016: the Korea National Health and Nutrition Examination Survey. Nutr Res Pract 2020;14:501-518.
8. Korea Centers for Disease Control and Prevention. Guideline for Nutrition Survey in Korea National Health and Nutrition Examination Survey 7th (2016-2018) [cited 2020 Sep 19]. Available from https://knhanes.kdca.go.kr/knhanes/sub04/sub04_02_02.do?classType=4 (Korean).
9. Korean Statistical Information Service. The ratio of 1 person household [cited 2020 Dec 22]. Available from: https://kosis.kr/statHtml/statHtml.do?orgId=101&tblId=DT_1YL21161&conn_path=I2 (Korean).
10. Korean Statistical Information Service. Labor force [cited 2020 Dec 22]. Available from: https://kosis.kr/statHtml/statHtml.do?orgId=101&tblId=DT_1DA7001S&conn_path=I2 (Korean).
11. U.S. Department of Agriculture. Scientific Report of the 2015 Dietary Guidelines Advisory Committee: Advisory Report to the Secretary of Health and Human Services and the Secretary of Agriculture; 2015 [cited 2020 Dec 22]. Available from: https://health.gov/sites/default/files/

2019-09/Scientific-Report-of-the-2015-Dietary-Guidelines-Advisory-Committee.pdf.

1. Marriott BP, Hunt KJ, Malek AM, and Newman JC. Trends in Intake of Energy and Total Sugar from Sugar-Sweetened Beverages in the United States among Children and Adults, NHANES 2003–2016. Nutrients 2019;11:2004.
2. Saito A, Imai S, Htun NC, Okada E, Yoshita K, Yoshiike N, et al. The trends in total energy, macronutrients and sodium intake among Japanese: findings from the 1995–2016 National Health and Nutrition Survey. Br J Nutr 2018;120:424–434.
3. Shan Z, Rehm CD, Rogers G, Ruan M, Wang DD, Hu FB, et al. Trends in dietary carbohydrate, protein, and fat intake and diet quality among US adults, 1999-2016. JAMA 2019;322:1178-1187.
4. Ministry of Health and Welfare. Report on 1998 National Health and Nutrition Survey (dietary intake survey). Seoul: Ministry of Health and Welfare; 2000, p. 674 (Korean).
5. Park HK, Lee Y, Kang BW, Kwon KI, Kim JW, Kwon OS, et al. Progress on sodium reduction in South Korea. BMJ Glob Health 2020;5:e002028.
6. National Institute of Agricultural Sciences. Food Composition Table. version 9.1 [cited 2020 Sep 19]. Available from http://koreanfood.rda.go.kr/kfi/fct/fctFoodSrch/list (Korean).
7. National Academy of Agricultural Sciences. Food Composition Table. version 8. Paju: Gyomoonsa; 2012, p.1-640 (Korean).

**Table 1. Characteristics of subjects in the Korea National Health and Nutrition Examination Survey**

|  | **1998** | | **2001** | | **2005** | | **2007** | | **2008** | | **2009** | | | **2010** | | | **2011** | | | **2012** | | | **2013** | | | **2014** | | | **2015** | | | **2016** | | | **2017** | | | **2018** | |
| --- | --- | --- | --- | --- | --- | --- | --- | --- | --- | --- | --- | --- | --- | --- | --- | --- | --- | --- | --- | --- | --- | --- | --- | --- | --- | --- | --- | --- | --- | --- | --- | --- | --- | --- | --- | --- | --- | --- | --- |
| **Total (n)** | 10,400 | | 9,968 | | 8,930 | | 4,091 | | 8,631 | | 9,391 | | | 8,019 | | | 7,704 | | | 7,208 | | | 7,242 | | | 6,801 | | | 6,628 | | | 7,040 | | | 7,167 | | | 7,064 | |
| Age (yr; Mean, SE) | 34.5 | 0.2 | 34.0 | 0.2 | 36.1 | 0.2 | 38.2 | 0.4 | 38.4 | 0.2 | 39.1 | 0.2 | 39.4 | | 0.3 | 41.3 | | 0.3 | 42.5 | | 0.3 | 40.0 | | 0.3 | 42.6 | | 0.3 | 43.6 | | 0.3 | 41.5 | | 0.3 | 43.4 | | 0.3 | 43.5 | | 0.3 |
| Age (yr; %, SE) |  |  |  |  |  |  |  |  |  |  |  |  |  | |  |  | |  |  | |  |  | |  |  | |  |  | |  |  | |  |  | |  |  | |  |
| 1-18 | 27.9 | 0.4 | 28.9 | 0.5 | 26.9 | 0.5 | 28.2 | 0.7 | 27.3 | 0.5 | 25.7 | 0.5 | 25.9 | | 0.5 | 23.6 | | 0.5 | 22.8 | | 0.5 | 24.9 | | 0.5 | 21.7 | | 0.5 | 19.9 | | 0.5 | 22.9 | | 0.5 | 20.2 | | 0.5 | 19.3 | | 0.5 |
| 19-64 | 62.8 | 0.5 | 62.0 | 0.5 | 61.8 | 0.5 | 54.5 | 0.8 | 55.8 | 0.5 | 57.0 | 0.5 | 56.8 | | 0.6 | 57.1 | | 0.6 | 55.2 | | 0.6 | 57.2 | | 0.6 | 56.0 | | 0.6 | 58.1 | | 0.6 | 56.8 | | 0.6 | 58.3 | | 0.6 | 59.6 | | 0.6 |
| 65+ | 9.4 | 0.3 | 9.2 | 0.3 | 11.2 | 0.3 | 17.4 | 0.6 | 16.9 | 0.4 | 17.3 | 0.4 | 17.3 | | 0.4 | 19.3 | | 0.4 | 22.0 | | 0.5 | 17.9 | | 0.5 | 22.2 | | 0.5 | 22.0 | | 0.5 | 20.4 | | 0.5 | 21.6 | | 0.5 | 21.1 | | 0.5 |
| Residence (%, SE) |  |  |  |  |  |  |  |  |  |  |  |  |  | |  |  | |  |  | |  |  | |  |  | |  |  | |  |  | |  |  | |  |  | |  |
| Urban | 63.4 | 0.5 | 79.0 | 0.4 | 80.5 | 0.4 | 72.0 | 0.7 | 75.7 | 0.5 | 76.0 | 0.4 | 79.1 | | 0.5 | 81.0 | | 0.4 | 80.6 | | 0.5 | 80.1 | | 0.5 | 80.3 | | 0.5 | 81.1 | | 0.5 | 80.7 | | 0.5 | 81.9 | | 0.5 | 82.4 | | 0.5 |
| Rural area | 36.6 | 0.5 | 21.0 | 0.4 | 19.5 | 0.4 | 28.0 | 0.7 | 24.3 | 0.5 | 24.0 | 0.4 | 20.9 | | 0.5 | 19.0 | | 0.4 | 19.4 | | 0.5 | 19.9 | | 0.5 | 19.7 | | 0.5 | 18.9 | | 0.5 | 19.3 | | 0.5 | 18.1 | | 0.5 | 17.6 | | 0.5 |
| Education (%, SE) |  |  |  |  |  |  |  |  |  |  |  |  |  | |  |  | |  |  | |  |  | |  |  | |  |  | |  |  | |  |  | |  |  | |  |
| ≤ Elementary school | - | - | - | - | 39.8 | 0.5 | 48.2 | 0.8 | 46.9 | 0.6 | 43.6 | 0.5 | 43.0 | | 0.6 | 40.9 | | 0.6 | 40.8 | | 0.6 | 39.8 | | 0.6 | 39.4 | | 0.6 | 37.0 | | 0.6 | 37.2 | | 0.6 | 35.7 | | 0.6 | 33.0 | | 0.6 |
| Middle school | - | - | - | - | 11.8 | 0.3 | 10.6 | 0.5 | 11.2 | 0.3 | 12.0 | 0.3 | 10.9 | | 0.4 | 11.7 | | 0.4 | 11.1 | | 0.4 | 11.2 | | 0.4 | 11.6 | | 0.4 | 11.6 | | 0.4 | 10.5 | | 0.4 | 10.8 | | 0.4 | 10.2 | | 0.4 |
| High school | - | - | - | - | 29.3 | 0.5 | 23.9 | 0.7 | 24.6 | 0.5 | 26.0 | 0.5 | 24.1 | | 0.5 | 25.4 | | 0.5 | 25.7 | | 0.5 | 26.2 | | 0.5 | 25.0 | | 0.6 | 26.5 | | 0.6 | 24.5 | | 0.5 | 24.2 | | 0.5 | 26.7 | | 0.5 |
| ≥ College | - | - | - | - | 19.0 | 0.4 | 17.2 | 0.6 | 17.3 | 0.4 | 18.4 | 0.4 | 22.0 | | 0.5 | 22.0 | | 0.5 | 22.4 | | 0.5 | 22.8 | | 0.5 | 24.1 | | 0.6 | 25.0 | | 0.6 | 27.8 | | 0.6 | 29.3 | | 0.6 | 30.1 | | 0.6 |
| **Male (n)** | 4,984 | | 4,760 | | 4,167 | | 1,821 | | 3,692 | | 4,182 | | | 3,550 | | | 3,376 | | | 3,127 | | | 3,196 | | | 2,976 | | | 2,942 | | | 3,063 | | | 3,233 | | | 3,144 | |
| Age (yr; Mean, SE) | 33.1 | 0.3 | 32.5 | 0.3 | 34.8 | 0.3 | 35.8 | 0.6 | 36.4 | 0.4 | 37.3 | 0.4 | 38.2 | | 0.4 | 39.6 | | 0.4 | 40.4 | | 0.4 | 38.3 | | 0.4 | 40.8 | | 0.4 | 42.0 | | 0.4 | 40.1 | | 0.4 | 41.9 | | 0.4 | 41.8 | | 0.4 |
| Age (yr; %, SE) |  |  |  |  |  |  |  |  |  |  |  |  |  | |  |  | |  |  | |  |  | |  |  | |  |  | |  |  | |  |  | |  |  | |  |
| 1-18 | 30.2 | 0.7 | 31.7 | 0.7 | 30.0 | 0.7 | 34.2 | 1.1 | 32.8 | 0.8 | 30.3 | 0.7 | 30.3 | | 0.8 | 28.2 | | 0.8 | 28.7 | | 0.8 | 29.1 | | 0.8 | 26.1 | | 0.8 | 23.6 | | 0.8 | 26.9 | | 0.8 | 23.2 | | 0.7 | 22.3 | | 0.7 |
| 19-64 | 62.2 | 0.7 | 60.9 | 0.7 | 60.4 | 0.8 | 49.8 | 1.2 | 51.8 | 0.8 | 53.6 | 0.8 | 52.6 | | 0.8 | 53.3 | | 0.9 | 49.8 | | 0.9 | 53.4 | | 0.9 | 51.8 | | 0.9 | 55.1 | | 0.9 | 52.5 | | 0.9 | 56.0 | | 0.9 | 58.3 | | 0.9 |
| 65+ | 7.6 | 0.4 | 7.4 | 0.4 | 9.6 | 0.5 | 16.0 | 0.9 | 15.3 | 0.6 | 16.1 | 0.6 | 17.2 | | 0.6 | 18.6 | | 0.7 | 21.5 | | 0.7 | 17.4 | | 0.7 | 22.1 | | 0.8 | 21.3 | | 0.8 | 20.7 | | 0.7 | 20.8 | | 0.7 | 19.4 | | 0.7 |
| Residence (%, SE) |  |  |  |  |  |  |  |  |  |  |  |  |  | |  |  | |  |  | |  |  | |  |  | |  |  | |  |  | |  |  | |  |  | |  |
| Urban | 64.1 | 0.7 | 79.1 | 0.6 | 80.2 | 0.6 | 71.2 | 1.1 | 75.2 | 0.7 | 75.8 | 0.7 | 79.2 | | 0.7 | 81.1 | | 0.7 | 80.1 | | 0.7 | 79.4 | | 0.7 | 79.2 | | 0.7 | 80.0 | | 0.7 | 80.2 | | 0.7 | 81.3 | | 0.7 | 82.4 | | 0.7 |
| Rural area | 35.9 | 0.7 | 20.9 | 0.6 | 19.8 | 0.6 | 28.8 | 1.1 | 24.8 | 0.7 | 24.2 | 0.7 | 20.8 | | 0.7 | 18.9 | | 0.7 | 19.9 | | 0.7 | 20.6 | | 0.7 | 20.8 | | 0.7 | 20.0 | | 0.7 | 19.8 | | 0.7 | 18.7 | | 0.7 | 17.6 | | 0.7 |
| Education (%, SE) |  |  |  |  |  |  |  |  |  |  |  |  |  | |  |  | |  |  | |  |  | |  |  | |  |  | |  |  | |  |  | |  |  | |  |
| ≤ Elementary school | - | - | - | - | 36.3 | 0.7 | 47.4 | 1.3 | 44.7 | 0.8 | 41.3 | 0.8 | 40.5 | | 0.9 | 38.4 | | 0.9 | 39.5 | | 0.9 | 38.5 | | 0.9 | 37.4 | | 1.0 | 35.0 | | 1.0 | 36.1 | | 0.9 | 33.1 | | 0.9 | 30.3 | | 0.9 |
| Middle school | - | - | - | - | 12.1 | 0.5 | 10.7 | 0.8 | 12.9 | 0.6 | 12.9 | 0.5 | 12.2 | | 0.6 | 12.4 | | 0.6 | 11.9 | | 0.6 | 11.4 | | 0.6 | 12.8 | | 0.7 | 11.4 | | 0.6 | 10.7 | | 0.6 | 11.4 | | 0.6 | 10.5 | | 0.6 |
| High school | - | - | - | - | 30.0 | 0.7 | 23.0 | 1.1 | 24.0 | 0.7 | 26.0 | 0.7 | 24.1 | | 0.8 | 25.5 | | 0.8 | 25.3 | | 0.8 | 26.4 | | 0.8 | 24.9 | | 0.9 | 28.0 | | 0.9 | 23.7 | | 0.8 | 25.0 | | 0.8 | 27.9 | | 0.8 |
| ≥ College | - | - | - | - | 21.6 | 0.6 | 18.9 | 1.0 | 18.4 | 0.7 | 19.8 | 0.6 | 23.2 | | 0.7 | 23.7 | | 0.8 | 23.2 | | 0.8 | 23.7 | | 0.8 | 24.9 | | 0.9 | 25.6 | | 0.9 | 29.5 | | 0.9 | 30.5 | | 0.9 | 31.4 | | 0.9 |
| **Female (n)** | 5,416 | | 5,208 | | 4,763 | | 2,270 | | 4,939 | | 5,209 | | | 4,469 | | | 4,328 | | | 4,081 | | | 4,046 | | | 3,825 | | | 3,686 | | | 3,977 | | | 3,934 | | | 3,920 | |
| Age (yr; Mean, SE) | 35.8 | 0.3 | 35.3 | 0.3 | 37.3 | 0.3 | 40.1 | 0.5 | 39.9 | 0.3 | 40.6 | 0.3 | 40.4 | | 0.3 | 42.6 | | 0.3 | 44.0 | | 0.4 | 41.3 | | 0.4 | 44.0 | | 0.4 | 44.8 | | 0.4 | 42.5 | | 0.4 | 44.6 | | 0.4 | 44.9 | | 0.4 |
| Age (yr; %, SE) |  |  |  |  |  |  |  |  |  |  |  |  |  | |  |  | |  |  | |  |  | |  |  | |  |  | |  |  | |  |  | |  |  | |  |
| 1-18 | 25.8 | 0.6 | 26.3 | 0.6 | 24.2 | 0.6 | 23.3 | 0.9 | 23.2 | 0.6 | 22.1 | 0.6 | 22.4 | | 0.6 | 20.1 | | 0.6 | 18.2 | | 0.6 | 21.5 | | 0.6 | 18.4 | | 0.6 | 17.0 | | 0.6 | 19.8 | | 0.6 | 17.7 | | 0.6 | 16.8 | | 0.6 |
| 19-64 | 63.2 | 0.7 | 62.9 | 0.7 | 63.1 | 0.7 | 58.2 | 1.0 | 58.8 | 0.7 | 59.7 | 0.7 | 60.2 | | 0.7 | 60.0 | | 0.7 | 59.4 | | 0.8 | 60.2 | | 0.8 | 59.3 | | 0.8 | 60.4 | | 0.8 | 60.1 | | 0.8 | 60.1 | | 0.8 | 60.7 | | 0.8 |
| 65+ | 11.0 | 0.4 | 10.8 | 0.4 | 12.6 | 0.5 | 18.5 | 0.8 | 18.1 | 0.5 | 18.2 | 0.5 | 17.4 | | 0.6 | 19.9 | | 0.6 | 22.3 | | 0.7 | 18.3 | | 0.6 | 22.4 | | 0.7 | 22.5 | | 0.7 | 20.1 | | 0.6 | 22.2 | | 0.7 | 22.5 | | 0.7 |
| Residence (%, SE) |  |  |  |  |  |  |  |  |  |  |  |  |  | |  |  | |  |  | |  |  | |  |  | |  |  | |  |  | |  |  | |  |  | |  |
| Urban | 62.8 | 0.7 | 78.9 | 0.6 | 80.8 | 0.6 | 72.6 | 0.9 | 76.1 | 0.6 | 76.2 | 0.6 | 79.1 | | 0.6 | 81.0 | | 0.6 | 80.9 | | 0.6 | 80.6 | | 0.6 | 81.2 | | 0.6 | 82.0 | | 0.6 | 81.1 | | 0.6 | 82.3 | | 0.6 | 82.4 | | 0.6 |
| Rural area | 37.2 | 0.7 | 21.1 | 0.6 | 19.2 | 0.6 | 27.4 | 0.9 | 23.9 | 0.6 | 23.8 | 0.6 | 20.9 | | 0.6 | 19.0 | | 0.6 | 19.1 | | 0.6 | 19.4 | | 0.6 | 18.8 | | 0.6 | 18.0 | | 0.6 | 18.9 | | 0.6 | 17.7 | | 0.6 | 17.6 | | 0.6 |
| Education (%, SE) |  |  |  |  |  |  |  |  |  |  |  |  |  | |  |  | |  |  | |  |  | |  |  | |  |  | |  |  | |  |  | |  |  | |  |
| ≤ Elementary school | - | - | - | - | 42.9 | 0.7 | 48.9 | 1.1 | 48.6 | 0.7 | 45.5 | 0.7 | 45.0 | | 0.8 | 42.8 | | 0.8 | 41.8 | | 0.8 | 40.9 | | 0.8 | 40.9 | | 0.9 | 38.5 | | 0.9 | 38.0 | | 0.8 | 37.7 | | 0.8 | 35.2 | | 0.8 |
| Middle school | - | - | - | - | 11.5 | 0.5 | 10.6 | 0.7 | 10.0 | 0.4 | 11.3 | 0.4 | 9.9 | | 0.5 | 11.1 | | 0.5 | 10.5 | | 0.5 | 11.1 | | 0.5 | 10.6 | | 0.5 | 11.7 | | 0.6 | 10.4 | | 0.5 | 10.3 | | 0.5 | 10.0 | | 0.5 |
| High school | - | - | - | - | 28.8 | 0.7 | 24.5 | 1.0 | 25.0 | 0.6 | 26.0 | 0.6 | 24.1 | | 0.7 | 25.3 | | 0.7 | 26.0 | | 0.7 | 26.0 | | 0.7 | 25.0 | | 0.8 | 25.4 | | 0.8 | 25.2 | | 0.7 | 23.6 | | 0.7 | 25.7 | | 0.7 |
| ≥ College | - | - | - | - | 16.8 | 0.5 | 16.0 | 0.8 | 16.4 | 0.5 | 17.2 | 0.5 | 21.0 | | 0.6 | 20.8 | | 0.6 | 21.7 | | 0.7 | 22.1 | | 0.7 | 23.4 | | 0.7 | 24.5 | | 0.8 | 26.5 | | 0.7 | 28.4 | | 0.8 | 29.1 | | 0.8 |

SE, standard error

**Table 2. Trends in the major food groups in the Korea National Health and Nutrition Examination Survey from 1998 to 2018**

(unit: g/d)

| Food groups | Sex | 1998 | | 2018 | | Diff. | APC | Significant change in the trend slope | | |
| --- | --- | --- | --- | --- | --- | --- | --- | --- | --- | --- |
|  |  | Mean(SE) | | Mean(SE) | |  |  | APC before the year of change | Year of change | APC after the year of change |
| Grains and cereals | Total | 337.2 | (3.3) | 288.4 | (2.8) | -48.8 | -0.4^*^ | -0.8 | 2007 | -0.2 |
|  | Male | 372.4 | (4.5) | 326.8 | (3.9) | -45.6 | -0.3 | -0.9 | 2005 | 0.0 |
|  | Female | 304.0 | (3.1) | 248.4 | (2.8) | -55.6 | -0.7^*^ | -1.0 | 2007 | -0.5 |
| Vegetables | Total | 287.8 | (4.3) | 248.1 | (2.8) | -39.7 | -0.8^*^ | 1.4 | 2005 | -1.5^*^ |
|  | Male | 319.7 | (5.2) | 283.1 | (4.0) | -36.6 | -0.7^*^ | 1.5 | 2005 | -1.4^*^ |
|  | Female | 257.9 | (4.1) | 212.5 | (3.4) | -45.4 | -1.0^*^ | 0.9 | 2005 | -1.6^*^ |
| Fruits | Total | 197.3 | (5.3) | 129.2 | (3.7) | -68.1 | -0.8 | -0.2 | 2016 | -13.3 |
|  | Male | 176.0 | (5.8) | 118.4 | (4.4) | -57.6 | -0.6 | 0.0 | 2016 | -13.4 |
|  | Female | 218.5 | (6.3) | 140.5 | (5.0) | -78.0 | -1.0 | -0.5 | 2016 | -12.9 |
| Non-alcoholic beverages | Total | 45.3 | (2.0) | 208.4 | (5.6) | 163.1 | 9.2^*^ | 3.7 | 2005 | 10.9^*^ |
|  | Male | 48.7 | (2.6) | 226.0 | (7.9) | 177.3 | 9.4^*^ | 10.1^*^ | 2016 | -0.5 |
|  | Female | 42.1 | (2.1) | 189.0 | (5.9) | 146.9 | 8.9^*^ | 1.9 | 2005 | 11.1^*^ |
| Meat | Total | 67.9 | (1.7) | 129.8 | (2.9) | 61.9 | 0.5^*^ | -0.1 | 2007 | 0.8^*^ |
|  | Male | 82.7 | (2.3) | 160.0 | (4.5) | 77.3 | 0.7^*^ | 0.8^*^ | 2015 | -1.1 |
|  | Female | 53.7 | (1.6) | 97.9 | (3.5) | 44.2 | 0.1 | -0.7 | 2008 | 0.6 |
| Milk and dairy products | Total | 79.6 | (2.6) | 118.3 | (3.1) | 38.7 | 2.2^*^ | 3.6^*^ | 2011 | -0.2 |
|  | Male | 79.5 | (2.9) | 118.5 | (3.9) | 39.0 | 2.4^*^ | 3.5^*^ | 2012 | -0.5 |
|  | Female | 79.4 | (3.0) | 118.1 | (4.1) | 38.7 | 2.0^*^ | 3.6^*^ | 2010 | -0.1 |
| Eggs | Total | 21.7 | (0.6) | 31.0 | (1.1) | 9.3 | 2.0^*^ | 1.5 | 2008 | 2.4^*^ |
|  | Male | 25.1 | (0.8) | 34.4 | (1.6) | 9.3 | 1.7^*^ | 1.7^*^ | 2016 | -0.5 |
|  | Female | 18.6 | (0.7) | 27.6 | (1.0) | 9.0 | 2.2^*^ | 1.3 | 2008 | 2.9^*^ |

SE, standard error; Diff., difference between the data from 1998 and 2018; APC, annual percent change.

The age-standardized mean and SE were calculated using the 2005 population projections for Korea.

* *p*<0.05

**Table 3. Energy intake trends in the Korea National Health and Nutrition Examination Survey from 1998 to 2018**

| Energy intake | | Sex | 1998 | | 2018 | | Diff. | APC | Significant change in the trend slope | | |
| --- | --- | --- | --- | --- | --- | --- | --- | --- | --- | --- | --- |
|  |  |  | Mean(SE) | | Mean(SE) | |  |  | APC before the year of change | Year of change | APC after the year of change |
| Energy intake (kcal/d) | | Total | 1,934.3 | (17.9) | 1,987.7 | (17.7) | 53.4 | 0.5^*^ | -0.1 | 2007 | 0.8* |
|  |  | Male | 2,152.5 | (22.8) | 2,301.5 | (26.1) | 149.0 | 0.7^*^ | 0.8* | 2015 | -1.1 |
|  |  | Female | 1,729.2 | (17.1) | 1,661.1 | (17.1) | -68.1 | 0.1 | -0.7 | 2008 | 0.6 |
| Percentage of energy from | Fat | Total | 17.9 | (0.2) | 22.6 | (0.2) | 4.7 | 1.1* | 0.0 | 2009 | 1.9^*^ |
|  |  | Male | 18.4 | (0.2) | 22.7 | (0.2) | 4.3 | 1.0* | 0.5 | 2009 | 1.5^*^ |
|  |  | Female | 17.4 | (0.2) | 22.4 | (0.2) | 5.0 | 1.3* | 0.2 | 2009 | 2.2^*^ |
|  | Carbohydrates | Total | 67.1 | (0.2) | 62.2 | (0.2) | -4.9 | -0.3^*^ | 0.0 | 2009 | -0.6^*^ |
|  |  | Male | 66.2 | (0.2) | 61.6 | (0.3) | -4.6 | -0.3^*^ | -0.1 | 2009 | -0.6^*^ |
|  |  | Female | 68.0 | (0.2) | 62.8 | (0.3) | -5.2 | -0.3^*^ | 0.0 | 2009 | -0.7^*^ |
|  | Protein | Total | 15.0 | (0.1) | 15.2 | (0.1) | 0.2 | -0.1 | -0.3^*^ | 2014 | 1.2 |
|  |  | Male | 15.4 | (0.1) | 15.7 | (0.1) | 0.3 | -0.1 | -0.3^*^ | 2014 | 1.4 |
|  |  | Female | 14.6 | (0.1) | 14.8 | (0.1) | 0.2 | -0.1 | -0.3^*^ | 2014 | 1.3 |
|  | Breakfast | Total | 23.1 | (0.2) | 16.2 | (0.3) | -6.9 | -1.8^*^ | -1.4^*^ | 2009 | -2.5^*^ |
|  |  | Male | 22.5 | (0.3) | 16.0 | (0.3) | -6.5 | -2.0^*^ | -1.3 | 2005 | -2.3^*^ |
|  |  | Female | 23.6 | (0.3) | 16.2 | (0.3) | -7.4 | -1.6^*^ | -1.0^*^ | 2009 | -2.5^*^ |
|  | Lunch | Total | 29.6 | (0.3) | 29.8 | (0.3) | 0.2 | -0.2 | -0.3^*^ | 2014 | 0.8 |
|  |  | Male | 30.0 | (0.3) | 29.0 | (0.4) | -1.0 | -0.2 | 0.2 | 2005 | -0.4 |
|  |  | Female | 29.4 | (0.3) | 30.7 | (0.4) | 1.3 | -0.1 | 0.7 | 2005 | -0.4 |
|  | Dinner | Total | 30.6 | (0.2) | 33.7 | (0.3) | 3.1 | 0.2^*^ | 0.0 | 2015 | 3.0^*^ |
|  |  | Male | 31.8 | (0.3) | 35.5 | (0.4) | 3.7 | 0.3^*^ | 0.1 | 2015 | 3.2^*^ |
|  |  | Female | 29.4 | (0.3) | 31.7 | (0.3) | 2.3 | 0.2 | -0.3 | 2012 | 1.4^*^ |
|  | Snacks | Total | 16.7 | (0.4) | 20.4 | (0.3) | 3.7 | 1.5^*^ | 2.6^*^ | 2014 | -3.1 |
|  |  | Male | 15.8 | (0.4) | 19.4 | (0.4) | 3.6 | 1.6^*^ | 2.7^*^ | 2014 | -3.8 |
|  |  | Female | 17.7 | (0.5) | 21.3 | (0.4) | 3.6 | 1.6^*^ | 2.4^*^ | 2015 | -4.0 |
|  | Homemade meal | Total | 65.5 | (0.7) | 38.4 | (0.6) | -27.1 | -2.7^*^ | -1.8^*^ | 2009 | -3.8^*^ |
|  |  | Male | 61.7 | (0.8) | 36.5 | (0.7) | -25.2 | -2.6^*^ | -1.8^*^ | 2009 | -3.7^*^ |
|  |  | Female | 69.2 | (0.8) | 40.4 | (0.7) | -28.8 | -2.7^*^ | -1.6^*^ | 2008 | -3.8^*^ |
|  | Dining out meal | Total | 18.9 | (0.4) | 36.6 | (0.6) | 17.7 | 2.4^*^ | 1.4 | 2011 | 4.0^*^ |
|  |  | Male | 23.1 | (0.5) | 39.7 | (0.8) | 16.6 | 2.0^*^ | 1.4 | 2012 | 3.5 |
|  |  | Female | 15.0 | (0.5) | 33.3 | (0.7) | 18.3 | 2.5^*^ | 0.9 | 2011 | 5.1^*^ |
|  | Convenience food | Total | 15.5 | (0.5) | 25.1 | (0.4) | 9.6 | 3.2^*^ | 5.3^*^ | 2012 | 0.1 |
|  |  | Male | 15.2 | (0.5) | 23.9 | (0.5) | 8.7 | 3.1^*^ | 4.4^*^ | 2012 | 0.8 |
|  |  | Female | 15.8 | (0.6) | 26.3 | (0.5) | 10.5 | 3.1^*^ | 5.1^*^ | 2013 | -1.2 |

SE, standard error; Diff., difference between the data from 1998 and 2018; APC, annual percent change.

The percentage of energy from fat means the percentage of energy from fat (g of fat × 9 kcal/g) compared to the sum of energy from fat, carbohydrates, and protein. The respective percentages of energy from the other components were calculated using a similar equation.

The age-standardized mean and SE were calculated using the 2005 population projections for Korea.

* *p*<0.05

**Table 4. Nutrient intake trends in the Korea National Health and Nutrition Examination Survey from 1998 to 2008**

| Nutrient | Sex | 1998 | | 2018 | | Diff. | APC | Significant change in the trend slope | | |
| --- | --- | --- | --- | --- | --- | --- | --- | --- | --- | --- |
|  |  | Mean | (SE) | Mean | (SE) |  |  | APC before the year of change | Year of change | APC after the year of change |
| Vitamin A  (㎍RE/d) | Total | 610.1 | (11.9) | 571.7 | (9.7) | -38.4 | -0.5 | 2.4^*^ | 2011 | -5.3^*^ |
|  | Male | 671.9 | (14.3) | 621.5 | (14.0) | -50.4 | -0.5 | 2.4^*^ | 2011 | -5.6^*^ |
|  | Female | 552.1 | (13.0) | 520.7 | (11.4) | -31.4 | -0.3 | 1.9^*^ | 2012 | -5.9^*^ |
| Riboflavin  (mg/d) | Total | 1.08 | (0.01) | 1.64 | (0.02) | 0.56 | 2.0^*^ | 0.6 | 2008 | 3.4^*^ |
|  | Male | 1.19 | (0.02) | 1.86 | (0.03) | 0.67 | 2.3^*^ | 0.4 | 2007 | 3.4^*^ |
|  | Female | 0.97 | (0.01) | 1.41 | (0.02) | 0.44 | 1.7^*^ | 0.0 | 2008 | 3.4^*^ |
| Vitamin C  (mg/d) | Total | 123.8 | (2.3) | 60.6 | (1.4) | -63.2 | -3.2^*^ | -1.9^*^ | 2014 | -11.7^*^ |
|  | Male | 122.3 | (2.5) | 65.3 | (2.2) | -57.0 | -3.0^*^ | -1.3^*^ | 2012 | -8.8^*^ |
|  | Female | 125.5 | (2.7) | 55.6 | (1.5) | -69.9 | -3.4^*^ | -2.3^*^ | 2015 | -15.3^*^ |
| Calcium  (mg/d) | Total | 500.7 | (7.5) | 516.2 | (6.3) | 15.5 | 0.1 | -0.1 | 2014 | 1.0 |
|  | Male | 541.4 | (9.5) | 571.6 | (8.1) | 30.2 | 0.2 | 0.1 | 2014 | 0.9 |
|  | Female | 462.0 | (7.1) | 459.2 | (7.4) | -2.8 | -0.1 | -0.3 | 2015 | 1.7 |
| Sodium  (mg/d) | Total | 4,585.6 | (62.4) | 3,255.0 | (37.4) | -1,330.6 | -2.3^*^ | -0.3 | 2010 | -4.9^*^ |
|  | Male | 5,130.5 | (73.4) | 3,809.5 | (51.7) | -1,321.0 | -1.9^*^ | 0.3 | 2010 | -5.0^*^ |
|  | Female | 4,068.7 | (63.1) | 2,679.6 | (37.9) | -1,389.1 | -2.8^*^ | 1.4 | 2005 | -4.1^*^ |
| Iron  (mg/d) | Total | 12.5 | (0.2) | 11.4 | (0.1) | -1.1 | -0.4 | 1.7^*^ | 2014 | -8.1^*^ |
|  | Male | 13.7 | (0.2) | 13.0 | (0.2) | -0.7 | 0.2 | 1.5^*^ | 2015 | -10.9^*^ |
|  | Female | 11.3 | (0.2) | 9.7 | (0.1) | -1.6 | -0.5 | 1.7^*^ | 2014 | -9.2^*^ |

SE, standard error; Diff., difference between the data from 1998 and 2018; APC, annual percent change.

The age-standardized mean and SE were calculated using the 2005 population projections for Korea.

* *p*<0.05
